# Physical exercise mitigates chronic psychological stress‐induced vascular inflammation via the BDNF–Kif4–TARM1 axis

**DOI:** 10.1002/ctm2.70674

**Published:** 2026-04-20

**Authors:** Xianghui Zheng, Yunqi Li, Peiyao Wang, Zhou Guo, Yuxuan Liu, Qi Liu, Baitao Wang, Huiyu Wang, Lizhi Zheng, Cien Li, Shuhong Liu, Shiyu Wang, Xinyu Hou, Xiaojun Wu, Yong Sun, Bo Yu, Yang Zheng, Jian Wu

**Affiliations:** ^1^ Department of Cardiology The Second Affiliated Hospital of Harbin Medical University Harbin China; ^2^ The Key Laboratory of Myocardial Ischemia Chinese Ministry of Education Harbin China; ^3^ State Key Laboratory of Frigid Zone Cardiovascular Diseases (SKLFZCD) Harbin Medical University Harbin China

**Keywords:** BDNF–Kif4–TARM1 axis, exercise, neuroimmune crosstalk, psychological stress, vascular inflammation

## Abstract

**Background:**

Chronic psychological stress drives neuroimmune crosstalk and accelerates atherosclerosis progression. Physical exercise confers broad health benefits and is associated with reduced inflammation. However, the exercise‐mediated factors and mechanisms that mitigate stress‐induced vascular inflammation remain unclear.

**Methods:**

Chronic restraint stress (CRS) and voluntary exercise models were established to investigate the role of exercise in neuroimmune crosstalk. RNA sequencing identified kinesin family member 4 (Kif4) as a key gene associated with the attenuation of stress‐induced inflammatory responses in peripheral blood monocytes following exercise. Combined co‐immunoprecipitation–mass spectrometry and membrane proteomics identified T cell‐interacting activating receptors on myeloid cell 1 (TARM1) as the Kif4 cargo. The function of TARM1 was validated using an immobilized TARM1‐Fc fusion protein. Brain‐derived neurotrophic factor (BDNF), a key effector during exercise and stress, regulated the Kif4–TARM1 axis using recombinant BDNF (rBDNF) and the TrkB inhibitor ANA‐12. Finally, exercise‐mediated effects and mechanisms were examined in atherosclerotic CRS‐exposed mouse models and in patients with coronary artery disease (CAD) experiencing high psychological stress.

**Results:**

Physical exercise alleviated stress‐induced neuroimmune crosstalk, reduced the proinflammatory CD11b^+^Ly6C^high^ monocyte phenotype, and suppressed M1 macrophage polarization. Kif4 knockdown mitigated proinflammatory responses in peripheral blood monocytes and BMDMs in the CRS model mice. Mechanistically, kinesin Kif4 facilitates microtubule‐dependent transport of TARM1 to the plasma membrane, thereby promoting macrophage inflammatory responses and enhancing monocyte‒endothelial cell adhesion. Conversely, neurogenic BDNF, regulated by exercise and stress, activated the TrkB receptor in monocytes/macrophages and inhibited the p‐STAT3/Kif4/TARM1 signalling pathway. Furthermore, in both an atherosclerotic mouse model and patients with CAD, exercise mitigated stress‐induced inflammation via the BDNF–Kif4–TARM1 axis.

**Conclusions:**

Physical exercise alleviates stress‐induced neuroimmune crosstalk through the BDNF–Kif4–TARM1 axis, revealing a novel neuroimmune‐mediated brain–heart axis that supports exercise‐based therapeutic strategies for psychogenic CAD.

**Key Points:**

- Chronic psychological stress drives systemic inflammation through neuroimmune mechanisms, thereby accelerating the progression of coronary artery disease (CAD).
- Physical exercise alleviates stress‐induced neuroimmune crosstalk, partly by suppressing proinflammatory responses in monocytes/macrophages.
- This study provides novel insights into exercise‐regulated neuroimmune mechanisms involving the monocyte BDNF–Kif4–TARM1 axis.
- In both an atherosclerotic mouse model and patients with CAD, exercise mitigated stress‐induced inflammation via the BDNF–Kif4–TARM1 axis.

## Graphical abstract

Schematic model of the potential mechanisms by which physical exercise alleviates stress‐induced vascular inflammation via the BDNF–Kif4–TARM1 axis, revealing a neuroimmune‐mediated brain–heart axis that supports exercise therapy for psychogenic coronary artery disease. This graphical abstract was drawn with the FigDraw tool (www.fgdraw.com).

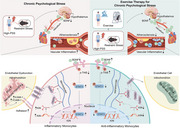

## 1 INTRODUCTION

Chronic psychological stress, defined as the body's response to common environmental challenges that exceed an individual's coping capacity, has detrimental effects on human health.^1^ Clinical and experimental studies have linked stress to the development of various diseases, particularly cardiovascular and immune diseases.^2^ The INTERHEART study, a large case‒control analysis conducted across 52 countries, identified psychological stress as a significant cardiovascular risk factor, reporting an odds ratio of 2.67 for myocardial infarction (MI).^3^ Furthermore, stress at work and in private life has been associated with a 40–50% increased risk of coronary artery disease (CAD) in prospective cohort studies.^4^ The pathophysiological mechanisms by which chronic psychological stress promotes atherosclerosis progression are multifactorial and include autonomic nervous dysfunction, enhanced inflammatory responses, altered signalling pathways, disrupted lipid metabolism and vascular endothelial dysfunction.^5^ Therefore, the mechanisms underlying psychogenic CAD and potential intervention strategies are of significant interest.

The immune system critically plays a central role in linking psychological stress to cardiovascular pathogenesis.^6^ Immunity and inflammation contribute to all stages of atherosclerosis.^7^ Chronic psychological stress increases circulating levels of leukocytes (e.g., inflammatory monocytes and neutrophils) and cytokines (e.g., interleukin‐6 and interleukin‐1β) through neuroimmune mechanisms.^8^, ^9^ This process promotes systemic inflammation and accelerates atherosclerosis progression.^10^ Although some small studies have suggested that interventions such as exercise or relaxation may reduce stress‐induced proinflammatory responses,^11^, ^12^ more studies are needed to understand the mechanisms underlying stress‐induced vascular inflammation and to develop appropriate treatment strategies.

Physical exercise is a potent, natural and practical therapy widely recognized for its broad health benefits.^13^ Exercise has been shown to benefit patients with stress‐induced CAD and anxiety disorders, possibly through anti‐inflammatory mechanisms.^14^ Specifically, voluntary exercise in mouse models reduces inflammatory cell production and cardiovascular inflammation.^15^ Physical exercise also reduces stress‐induced amygdala activity and disrupts its association with systemic inflammation in obese women, which explains the mechanism underlying the beneficial health effect of exercise on cardiovascular disease via the attenuation of stress neurobiology.^16^ Moreover, exercise increases circulating levels of brain‐derived neurotrophic factor (BDNF),^17^ which is primarily derived from the brain,^18^ and further attenuates ejection fraction reduction post‐MI and neuroinflammation.^19^ Collectively, exercise has therapeutic potential for combating cardiovascular disease resulting from chronic psychological stress.

However, the effects of exercise on stress‐induced neuroimmune crosstalk remain incompletely understood, and the molecular mediators of its anti‐inflammatory effects remain unclear. In this study, we demonstrate that physical exercise alleviates the stress‐induced vascular inflammation through the BDNF–Kif4–TARM1 axis of monocytes/macrophages, revealing a neuroimmune‐mediated brain–heart axis that supports exercise therapy for psychogenic CAD.

## 2 METHODS

All detailed methodological information is available in the Supporting Information material.

### 2.1 Animals and participants

Male C57BL/6J mice (6–8 weeks old) were obtained from the Experimental Animal Center of the Second Affiliated Hospital of Harbin Medical University. Male ApoE^−/−^ mice (8 weeks old) were sourced from Beijing Vital River Laboratory Animal Technology Co., Ltd. Atherosclerosis was induced in ApoE^−/−^ mice by feeding them a high‐fat diet (HFD) for 12 weeks. All mice were housed under controlled environmental conditions (23°C, 50–60% humidity, 12 h light/dark cycle) at the Experimental Animal Center of the Second Affiliated Hospital of Harbin Medical University. All animal experiments were performed in accordance with the National Institutes of Health guidelines for the care and use of laboratory animals. Mice were anaesthetized by isoflurane inhalation (1.5–2%) and euthanized by cervical dislocation before tissue collection. Patients with chronic coronary syndrome (CCS) secondary to CAD who were receiving routine treatment and had no severe liver or kidney diseases, malignancy, severe autoimmune diseases or infectious diseases were enrolled during their routine 3‐month post‐discharge outpatient follow‐up visits at the Second Affiliated Hospital of Harbin Medical University between September and December 2023. Patients were divided into four groups (*n* = 30 per group) based on the 10‐item perceived stress scale (PSS‐10) scores (low < 14 points; high ≥ 14 points) and regular exercise habits (defined as aerobic exercise performed 3–5 times per week). Whole blood samples were collected from all participants. The study was conducted in accordance with the Declaration of Helsinki. All participants provided written informed consent. The study protocol was approved by the Ethics Committee of the Second Affiliated Hospital of Harbin Medical University (approval no. YJSDW2023‐250).

### 2.2 Mouse model

For the chronic stress restraint (CRS) model, mice were subjected to daily restraint in a perforated plastic tube (10 cm length × 2.5 cm diameter; ventilation holes at the head end) for 2 h (18:00–20:00) over 14 consecutive days.^20^ In the exercise paradigm, mice in the voluntary running group performed 1 h of wheel running exercise (21:00 to 22:00) daily for 14 consecutive days.^21^ The wheel had an inner diameter of 20 cm, with a magnetic sensor installed at the top and a magnet attached to record the spontaneous movement and running distance of the mouse.

### 2.3 Behavioural tests

The open field test (OFT) was conducted in a plastic chamber (50 × 50 × 40 cm) under overhead lighting. The central zone was defined as a 25 × 25 cm area. At the beginning of the experiment, each mouse was gently placed in the centre of the chamber and allowed to freely explore the arena for 5 min. Movement paths were captured using a camera and analyzed using the SMART 3.0 animal behaviour video analysis system (Panlab, Spain) at the Harbin Institute of Technology to obtain the time spent in the central zone and the total movement distance. For the elevated plus maze (EPM) test, mice were placed on a central platform (5 cm × 5 cm) and allowed to explore freely for 5 min. The maze consisted of two opposing open arms (30 cm × 5 cm × 0.5 cm) and two opposing enclosed arms (30 cm × 5 cm × 15 cm). The time spent in the open and enclosed arms was recorded using the Animal Behavior Video Analysis System (SMART 3.0, Panlab) at the Harbin Institute of Technology.

### 2.4 Cell culture

Human monocyte cell line THP‐1, human umbilical vein endothelial cells (HUVECs) and RAW264.7 cells were purchased from the American Type Culture Collection. Kif4 knockout THP‐1 and RAW264.7 cells were purchased from Cyagen Biosciences and UBIGENE Biosciences, respectively. THP‐1 cells were cultured in RPMI 1640 medium (Gibco, Thermo Fisher Scientific) supplemented with 10% FBS and 1% penicillin/streptomycin. HUVECs were cultured in DMEM/F12 (Gibco, Thermo Fisher Scientific) supplemented with 10% FBS and 1% penicillin/streptomycin. RAW264.7 cells were cultured in Dulbecco's modified DMEM medium (Gibco, Thermo Fisher Scientific) supplemented with 10% fetal bovine serum (FBS) and 1% penicillin/streptomycin. All cells were cultured at 37°C with 5% CO_2_ and 95% humidity. THP‐1 cells and HUVECs were retrieved from liquid nitrogen storage and rapidly thawed in a 37°C water bath for 2–3 min until they were fully defrosted. The cell suspension was centrifuged at 1000 rpm for 5 min, after which the supernatant was discarded. The pellet was resuspended in 3 mL of complete culture medium. The resuspended cells were transferred to culture dishes and maintained at 37°C in a humidified incubator with 5% CO_2_ until they reached 90% confluency. The cells were subsequently passaged at a 1:3 ratio in new culture dishes for continued expansion.

### 2.5 Flow cytometry analysis

THP‐1 cells were seeded in six‐well plates (1 × 10^6^ cells/mL) and cultured with 100 ng/mL PMA (Beyotime) for 48 h, followed by LPS (500 ng/mL; Sigma‐Aldrich) for 24 h. Cells were harvested and stained with PE‐CD86 and APC‐CD163 antibodies (BioLegend) for 30 min at 4°C in the dark. Cells were washed, fixed and permeabilized using Fix/Perm buffer (Invitrogen, Thermo Fisher Scientific). FITC‐CD68 (BioLegend) was added to the cells for 30 min at room temperature. BMDMs and RAW264.7 cells were stimulated with LPS (500 ng/mL). The cells were harvested 24 h after stimulation and stained with PE‐conjugated anti‐CD86 and FITC‐conjugated anti‐F4/80 antibodies (BioLegend) for 30 min at 4°C in the dark to detect M1 macrophages. For M2 macrophage detection, the BMDMs were stained with a PE‐F4/80 antibody (BioLegend) for 30 min at 4°C in the dark. The cells were washed, fixed and permeabilized using Fix/Perm buffer (Invitrogen, Thermo Fisher Scientific), followed by incubation with an APC‐conjugated anti‐CD206 antibody (Invitrogen, Thermo Fisher Scientific) for 30 min at room temperature. The peripheral blood monocytes of the mice were stained with FITC‐conjugated anti‐CD45, PE‐conjugated anti‐Ly6C and APC‐conjugated anti‐CD11b antibodies (BioLegend) for 30 min at 4°C in the dark. Human peripheral blood monocytes were stained with PE‐conjugated anti‐CD14 and FITC‐conjugated anti‐CD16 antibodies (BioLegend) for 30 min at 4°C in the dark. Data were analyzed using FACSDiva version 6.1.3 (BD Biosciences) and FlowJo_V10 (TreeStar).

### 2.6 RNA sequencing (RNA‐seq)

RNA‐seq was performed by Shanghai Jiayin Biotechnology. Total RNA was extracted from peripheral blood monocytes. Libraries were prepared from polyA‐selected RNA and sequenced on the Illumina NovaSeq 6000 platform in high‐output mode as 150 bp paired‐end reads. Raw sequence files were processed and converted into sequence data using CASAVA base recognition and then converted to FASTQ format using in‐house Perl scripts. Sequence data were annotated using the ENSEMBL reference genome. The R package DESeq2 was used for gene expression analysis.

### 2.7 Coimmunoprecipitation–mass spectrum analysis and liquid chromatography‒tandem mass spectrometry

Protein samples from the immunoprecipitation and IgG control groups, along with membrane proteome extracts of control and Kif4‐overexpressing (OE‐Kif4) BMDMs, were flash‐frozen in liquid nitrogen and transported to Wuhan MetWare Biotechnology Co., Ltd. for IP‐MS and liquid chromatography‒tandem mass spectrometry (LC–MS/MS) analyses. Samples were analyzed using a Thermo Fisher Scientific mass spectrometer. Raw MS data were subjected to quality assessment and processing, followed by bioinformatics analysis of differentially expressed proteins.

### 2.8 Statistical analysis

Data normality was assessed using the Shapiro–Wilk test. Normally distributed data are presented as the means ± standard deviation (SDs), whereas non‐normally distributed data are presented as medians (P25, P75). The R language and GraphPad Prism 9 software were used for statistical analyses. The data were analyzed using Student's *t*‐test for comparisons between two groups and one‐way ANOVA for comparisons among three or more groups. Continuous variables that were not normally distributed across multiple groups were analyzed using the Kruskal–Wallis test, followed by Dunn's test for post hoc comparisons. A *p*‐value < .05 was considered to indicate statistical significance.

## 3 RESULTS

### 3.1 Physical exercise ameliorates stress‐induced neuroimmune crosstalk

**FIGURE 1:**
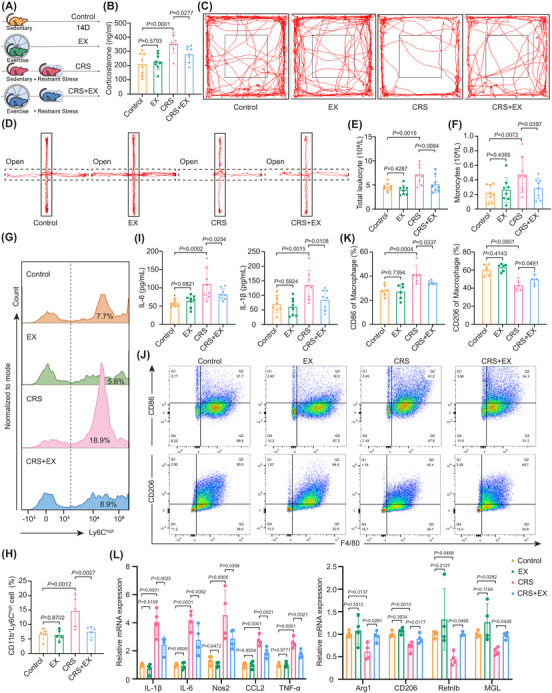
Voluntary exercise reduces stress‐induced anxiety‐like behaviours and systemic inflammation. (A) Experimental schematic. Male C57BL/6 wild‐type mice were exposed to CRS (2 h/day) and performed voluntary wheel running (1 h/day) for 14 days. (B) ELISA was performed to measure plasma corticosterone levels (*n* = 8). (C, D) Representative locomotion tracks of mice in the OFT and EPM tests are shown. (E, F) Complete blood cell count in peripheral blood, including the numbers of leukocytes and monocytes (*n* = 8). (G, H) Flow cytometry analysis of peripheral blood monocyte phenotypes. Representative flow cytometry plots and quantification of CD11b^+^Ly6C^high^ monocytes (*n* = 5). (I) Plasma concentrations of IL‐6 and IL‐1β quantified by ELISA (*n* = 8). (J, K) Flow cytometry analysis of BMDM polarization. Gating and quantification of M1 (F4/80^+^CD86^+^) and M2 (F4/80^+^CD206^+^) macrophages (*n* = 6). (L) qRT‐PCR analysis of proinflammatory (IL‐6, IL‐1β, Nos2, CCL2 and TNF‐α) and anti‐inflammatory (Arg1, CD206, Retnlb and MGL) gene expression in BMDMs (*n* = 4). The data are presented as the means ± SDs. *p‐*values were determined using one‐way ANOVA.

Given the well‐established anti‐inflammatory effects of exercise, we investigated whether exercise alleviates stress‐induced immune responses. Voluntary running and CRS models were established in C57BL/6 mice (Figure 1A). After 14 days of restraint stress, mice exhibited anxiety‐ and depression‐like behaviours in both the OFT and EPM. Specifically, the time spent in the central zone of the OFT and in the open arms of the EPM was significantly reduced (Figure 1C–D; Figure S1A–C). These behavioural alterations were accompanied by increased corticosterone levels (Figure 1B). Exercise intervention significantly reduced corticosterone levels in CRS‐exposed mice (Figure 1B). Concurrently, the time spent in the central zone of the open field (Figure 1C; Figure S1B) and in the open arms of the EPM increased significantly (Figure 1D; Figure S1C). These findings are consistent with previous findings^20^ and indicate that exercise effectively mitigates CRS‐induced anxiety‐ and depression‐like behaviours. Notably, after physical exercise and stress exposure, the body weight decreased (Figure S1D), but the exercise distance did not differ (Figure S1E).

Given the importance of blood leukocytes and monocytes in stress‐induced immune responses,^8^ we analyzed the numbers of blood leukocytes and monocytes. Total leukocyte and monocyte counts increased after CRS (Figure 1E,F), specifically the proportion of inflammatory CD11b^+^Ly6C^high^ monocytes (Figure S1F; Figure 1G,H). Exercise mitigated these changes. Furthermore, plasma levels of the proinflammatory cytokines IL‐6 and IL‐1β decreased in exercise‐treated CRS‐exposed mice (Figure 1I). Similarly, a reduction in the proportion of proinflammatory M1 macrophages in BMDM was observed, which was consistent with the findings in peripheral blood monocytes after exercise (Figure 1J,K). Here, increases in the expression of M2 macrophage genes (Arg1, CD206, Retnlb and MGL) and decreases in the expression of M1 macrophage genes (IL‐1β, IL‐6, Nos2, CCL2 and TNF‐α) were detected in exercise‐treated CRS‐exposed mice (Figure 1L). Collectively, these data indicate that physical exercise alleviates stress‐induced neuroimmune crosstalk.

### 3.2 Exercise attenuates stress‐induced inflammatory monocytes by suppressing the kinesin Kif4

**FIGURE 2:**
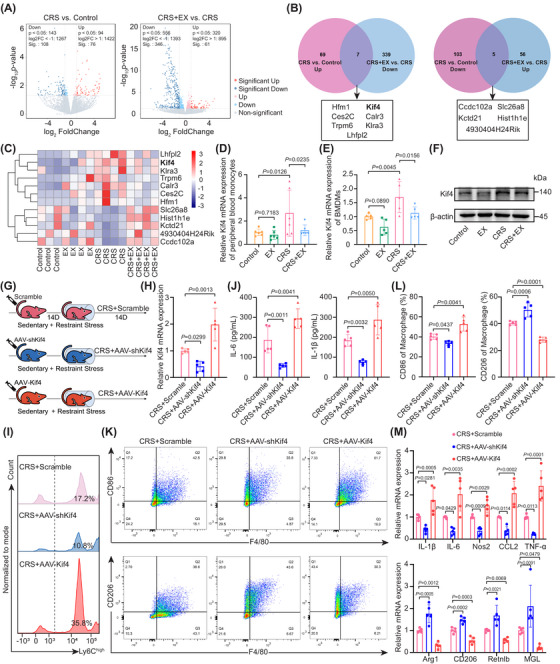
Targeting Kif4 enables the exercise‐mediated attenuation of stress‐induced inflammatory monocytes. (A) Volcano plots of DEGs for the CRS vs. control and CRS+EX vs. CRS comparisons; (B) Venn diagrams of overlapping DEGs: upregulated DEGs in the CRS vs. control group and downregulated DEGs in the CRS+EX vs. CRS group (left panel); downregulated DEGs in the CRS vs. control group and upregulated DEGs in the CRS+EX vs. CRS group (right panel). (C) Heatmap showing overlapping DEGs. (D, E) qRT‐PCR analysis of Kif4 expression in peripheral blood monocytes (*n* = 6) and BMDMs (*n* = 5). (F) Western blot analysis of Kif4 protein expression in peripheral blood monocytes. (G) Experimental schematic. Wild‐type mice received tail vein injections of scrambled plasmid, AAV‐shKif4, or AAV‐Kif4. Fourteen days after the injection, mice in all groups were subjected to daily restraint stress (2 h/day) for 14 consecutive days. (H) qRT‐PCR analysis of Kif4 expression in peripheral blood monocytes (*n* = 5). (I) Flow cytometry analysis of CD11b^+^Ly6C^high^ monocytes in peripheral blood. (J) Plasma IL‐6 and IL‐1β levels measured by ELISA (*n* = 5). (K, L) Gating strategy and quantification of BMDMs (*n* = 5). (M) qRT‐PCR analysis of inflammatory and anti‐inflammatory gene expression in BMDMs (*n* = 5). The data are presented as the means ± SDs. *p*‐values were determined using one‐way ANOVA.

To investigate the molecular basis underlying the reduction in inflammatory monocytes in exercise‐treated CRS‐exposed mice, we performed RNA‐seq on peripheral blood monocytes. Stress significantly upregulated Hfm1, Kif4, Ces2C, Calr3, Trpm6, Klra3 and Lhfpl2 expression, while downregulating Ccdc102a, Slc26a8, Kctd21, Hist1h1e and 4930404H24Rik expression. Exercise reversed these stress‐induced transcriptional changes (Figure 2A–C). Interestingly, kinesin family member 4 (Kif4) and Lhfpl2 have been reported to induce inflammatory monocytes.^22^, ^23^ In CRS‐exposed mice subjected to exercise intervention, Kif4 expression was reduced in both peripheral blood monocytes and BMDMs (Figure 2D–F; Figure S2C), whereas Lhfpl2 expression was not significantly altered (Figure S2A,B). Next, we investigated whether the kinesin Kif4 could regulate inflammatory responses in Kif4 knockout RAW264.7 cells and THP‐1 cells (Figure S2D,E). Flow cytometry showed a decreased proportion of the pro‐inflammatory M1 phenotype in both Kif4 knockout RAW264.7 and THP‐1 cells (Figure S2F,G,I,J). Correspondingly, qRT‐PCR indicated reduced expression in both cell lines, with IL‐1β, IL‐6, Nos2, CCL2 and TNF‐α in RAW264.7 cells and IL‐1β and TNF‐α in THP‐1 cells (Figure S2H,K).

Based on these findings, we further investigated whether Kif4 could regulate inflammatory responses in CRS‐exposed mice and exercise‐treated CRS‐exposed mice (Figure 2G; Figure S3A). After screening and validating the Kif4 plasmid (Figure S2L–O), we found that the injection of Kif4‐targeting AAV‐shRNA significantly reduced the proportion of CD11b^+^Ly6C^high^ monocytes compared with the injection of the scrambled plasmid in CRS‐exposed mice and exercise‐treated CRS‐exposed mice, supporting the proinflammatory role of Kif4 in mice subjected to stress (Figure 2H,I; Figure S3F,G; Figure S4E). Under the same stress modelling conditions, no differences in behavioural phenotypes were observed (Figure S3B–E; Figure S4A–D). Notably, consistent with the effects of exercise, ELISA revealed that Kif4 knockdown reduced the plasma levels of the proinflammatory cytokines IL‐6 and IL‐1β in both CRS‐exposed mice and exercise‐treated CRS‐exposed mice (Figure 2J; Figure S3H). Moreover, AAV‐shKif4 injection significantly reduced the proportion of proinflammatory M1 macrophages (Figure 2K,L; Figure S3I,J) and the overall expression of proinflammatory genes (IL‐1β, IL‐6, Nos2, CCL2 and TNF‐α) in BMDMs (Figure 2M; Figure S3K). Together, these results demonstrate that knockdown of kinesin Kif4 effectively antagonizes the stress‐induced inflammatory response in both peripheral blood monocytes and BMDMs, and that kinesin Kif4 may be a key regulator of neuroimmune crosstalk.

### 3.3 The Kif4–TARM1 axis drives macrophage inflammation in vitro

**FIGURE 3:**
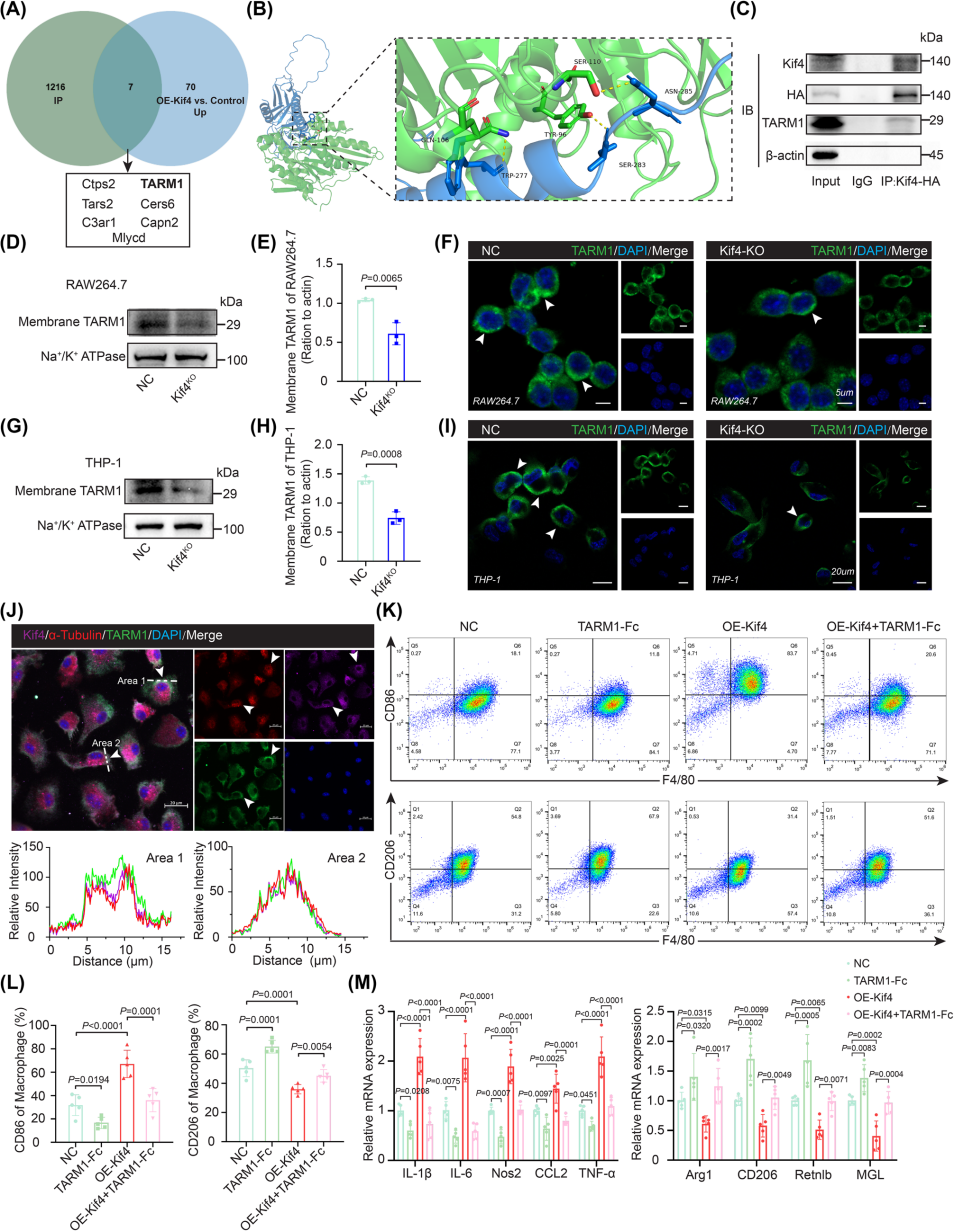
Kif4 promotes the microtubule‐dependent delivery of TARM1 to the plasma membrane, activating macrophage inflammation. (A) Venn diagram showing the overlap between Kif4‐interacting proteins in BMDMs and membrane proteins upregulated in OE‐Kif4 BMDMs compared with control BMDMs. (B) Schematic representation of molecular docking between the Kif4 (green) and TARM1 (blue) proteins, ΔiG = −10.4). (C) Co‐IP assay validating the interaction between Kif4 and TARM1. (D, E) Western blot analysis of TARM1 protein levels in membrane fractions isolated from RAW264.7 or Kif4 knockout RAW264.7 cells (*n* = 3). (F) Immunofluorescence staining showing that TARM1 (green) localized to the cell membrane in RAW264.7 or Kif4 knockout RAW264.7 cells. Scale bar = 5 µm. (G, H) Western blot analysis of TARM1 protein levels in membrane fractions isolated from THP‐1 or Kif4 knockout THP‐1 cells (*n* = 3). (I) Immunofluorescence staining showing that TARM1 (green) localized to the cell membrane in THP‐1 or Kif4 knockout THP‐1 cells. Scale bar = 20 µm. (J) Immunofluorescence staining showing Kif4 (purple), α‐tubulin (red; microtubules) and TARM1 (green) colocalization in BMDMs. Scale bar = 20 µm. (K, L) Flow cytometry gating strategy and quantification of M1 (F4/80^+^CD86^+^) and M2 (F4/80^+^CD206^+^) BMDMs transfected with TARM1‐Fc and/or OE‐Kif4 plasmids (*n* = 5). (M) qRT‐PCR analysis of inflammatory and anti‐inflammatory gene expression in BMDMs transfected with TARM1‐Fc or OE‐Kif4 plasmids (*n* = 5). The data are presented as the means ± SDs. *p*‐values were determined using two‐sample *t*‐tests or one‐way ANOVA.

Kif4 is a member of the kinesin family that plays an important role in protein transport. Since a previous study showed that Kif4 mediates hepatitis B virus/hepatitis delta virus entry by regulating the membrane protein sodium taurocholate‐cotransporting polypeptide (NTCP),^24^ we analyzed the proteome for proteins that specifically bind to Kif4 using coimmunoprecipitation–mass spectrum (CoIP–MS). Among the 1216 Kif4‐binding proteins, seven unique proteins (Ctps2, TARM1, Tars2, Cers6, C3ar1, Capn2 and Mlycd) were upregulated in the membrane proteome of OE‐Kif4 BMDMs compared with control BMDMs (Figure 3A). T cell‐interacting activating receptors on myeloid cell 1 (TARM1), known as inflammatory membrane protein receptors, play an important role in regulating the immune response of monocytes.^25^ Single‐cell RNA‐seq analysis of mouse atherosclerotic lesions (GSE239591) revealed that TARM1 is primarily expressed in immune cells, such as monocytes/macrophages, neutrophils and T cells (Figure S5A–C). Molecular docking further revealed that Kif4 formed three hydrogen bonds with TARM1 (Figure 3B). Co‐IP also confirmed the interaction between TARM1 and Kif4 (Figure 3C).

Thus, we examined the effect of Kif4 knockout on TARM1 expression. In both RAW264.7 and THP‐1 cells, Kif4 knockout led to the downregulation of membrane TARM1 (Figure 3D–I; Figure S5D,E). Consistent with this, downregulation of Kif4 in BMDMs altered TARM1 localization, decreasing its membrane levels while increasing those in the cytoplasm without affecting the total TARM1 protein (Figure S5F–M). This indicated that Kif4 influences the distribution, but not the overall expression, of TARM1. As kinesin Kif4 transports proteins along microtubules,^24^ we confirmed this function by immunofluorescence staining and observed the colocalization of Kif4, TARM1 and microtubules (Figure 3J). Furthermore, we investigated whether surface TARM1 activates macrophage inflammation. We first prepared an immobilized TARM1‐Fc fusion protein that binds to surface TARM1. Using flow cytometry, we found that TARM1‐Fc transfection into BMDMs suppressed M1 macrophage polarization (Figure 3K,L). Similarly, TARM1‐Fc treatment reduced the expression of M1 macrophage markers (IL‐1β, IL‐6, Nos2, CCL2 and TNF‐α) (Figure 3 M). Taken together, these results suggest that the Kif4–TARM1 axis is crucial for the attenuation of stress‐induced inflammatory macrophages following exercise.

### 3.4 TARM1 on the surface of monocytes promotes monocyte–endothelial cell adhesion, contributing to vascular inflammation

**FIGURE 4:**
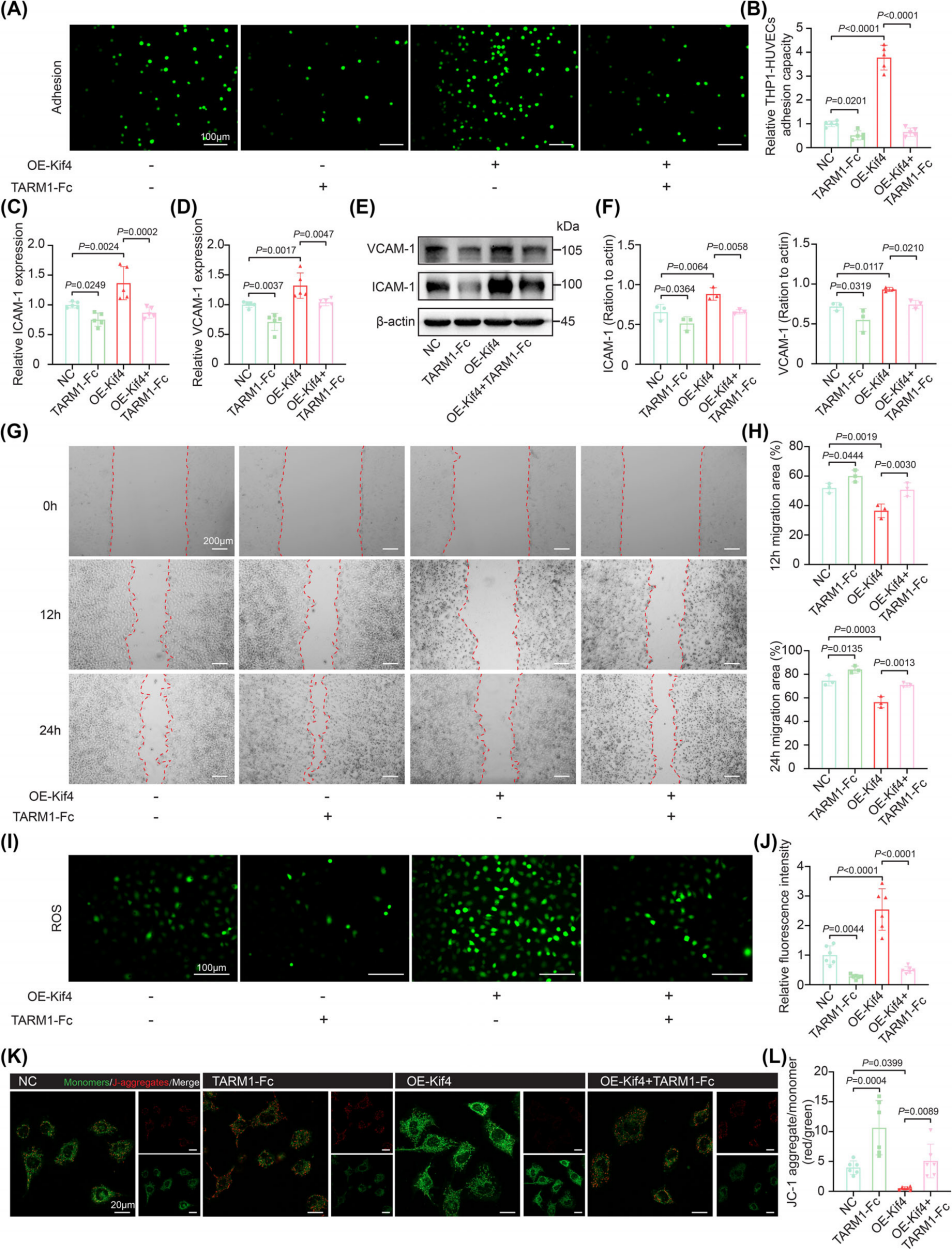
Monocyte surface TARM1 contributes to endothelial dysfunction. (A, B) Representative images of THP‐1 cells adhering to HUVECs and quantification of adhesion after 24 h of coculture with TARM1‐Fc‐ and/or OE‐Kif4‐treated THP‐1 cells (*n* = 5). Scale bar = 100 µm. (C, D) qRT‐PCR analysis of ICAM‐1 and VCAM‐1 expression in HUVECs following 24 h of coculture with TARM1‐Fc‐ and/or OE‐Kif4‐treated THP‐1 cells (*n* = 5). (E, F) Western blot analysis of ICAM‐1 and VCAM‐1 protein levels in HUVECs following 24 h of coculture with TARM1‐Fc‐ and/or OE‐Kif4‐treated THP‐1 cells (*n* = 3). (G, H) HUVEC migration following 12/24 h of coculture with TARM1‐Fc‐ or OE‐Kif4‐treated THP‐1 cells (*n* = 3). Scale bar = 200 µm. (I, J) Immunofluorescence staining for ROS in HUVECs after 24 h of coculture with TARM1‐Fc‐ and/or OE‐Kif4‐treated THP‐1 cells (*n* = 6). Scale bar = 100 µm. (K, L) JC‐1 staining and analysis of the mitochondrial membrane potential in HUVECs following 24 h of coculture with TARM1‐Fc‐ and/or OE‐Kif4‐treated BMDMs (*n* = 6). The data are presented as the means ± SDs. *p*‐values were determined using one‐way ANOVA.

Given the role of monocyte‐endothelial cell interactions in the development of vascular inflammation,^26^ we established a coculture system using THP‐1 cells treated with TARM1‐Fc and/or OE‐Kif4 and HUVECs. We found that monocyte‐endothelial cell adhesion increased between OE‐Kif4‐transfected THP‐1 cells and HUVECs after 24 h of coculture. Conversely, TARM1‐Fc treatment of these transfected cells attenuated intercellular adhesion (Figure 4A,B). The expression of intercellular cell adhesion molecule‐1 (ICAM‐1) and vascular cell adhesion protein 1 (VCAM‐1) in HUVECs further validated these findings (Figure 4C–F). Moreover, migration assays revealed that the migration ability of HUVECs was attenuated after 12/24 h of coculture with OE‐Kif4‐transfected THP‐1 cells. Conversely, the treatment of transfected cells with TARM1‐Fc enhanced HUVEC migration (Figure 4G,H).

Previous evidence indicates that endothelial cell mitochondrial damage is an important factor that promotes leukocyte (especially monocyte) adhesion.^27^ We next detected reactive oxygen species (ROS) levels and mitochondrial membrane potential in endothelial cells. The results showed that after 24 h of coculture, OE‐Kif4‐transfected THP‐1 cells showed increased ROS levels and a loss of mitochondrial membrane potential in HUVECs. In contrast, TARM1‐Fc treatment of these transfected cells attenuated mitochondrial damage in HUVECs (Figure 4I–L). Therefore, our findings indicate that surface TARM1 contributes to endothelial cell dysfunction in monocytes/macrophages, suggesting its role in vascular inflammation.

### 3.5 Exercise modulation of stress‐driven inflammatory monocytes depends on BDNF signalling suppressing the Kif4–TARM1 axis

**FIGURE 5:**
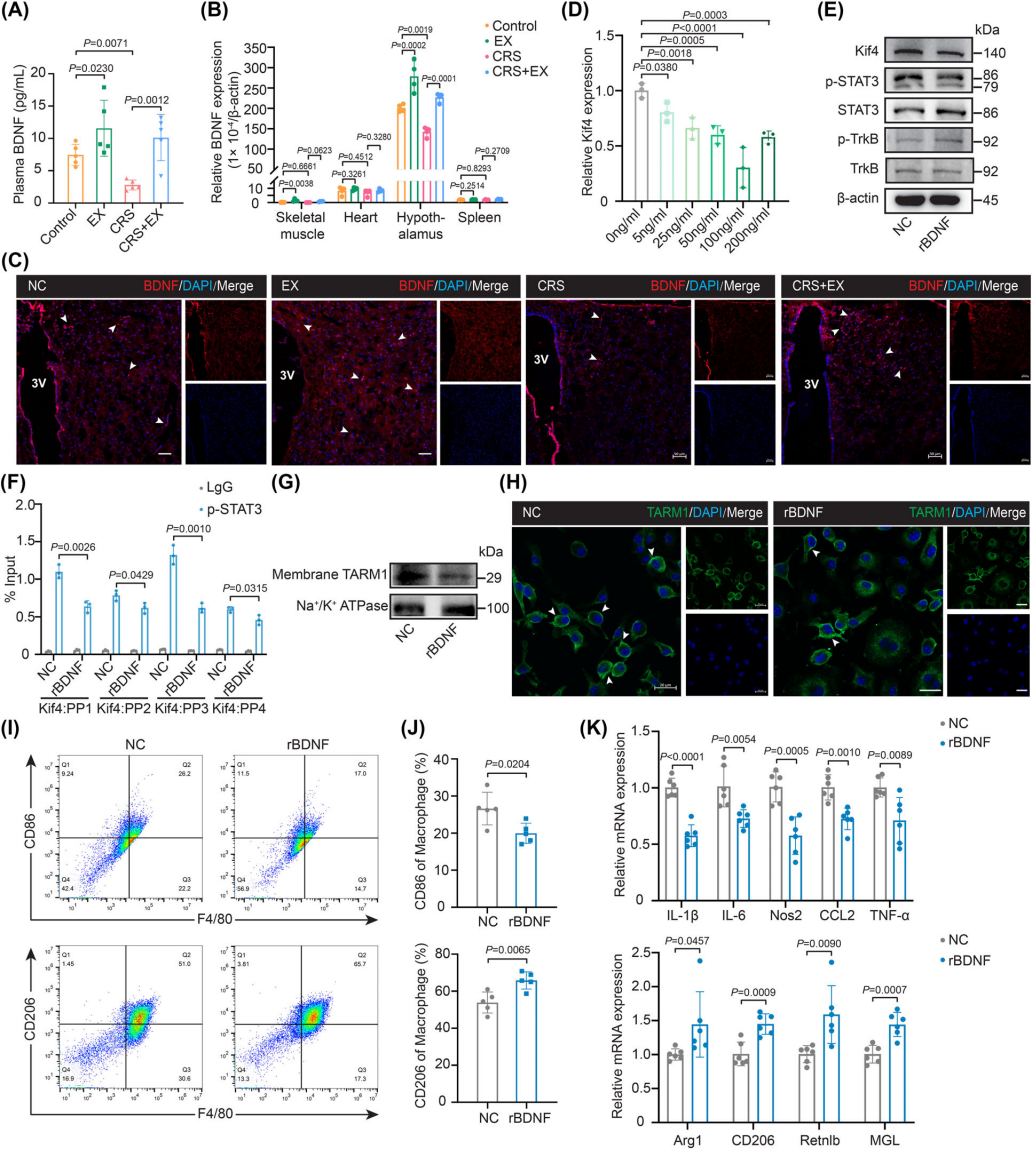
BDNF–TrkB–STAT3 signalling suppresses the Kif4–TARM1 axis to inhibit macrophage inflammation. (A) Plasma BDNF levels measured by ELISA in wild‐type mice after 14 days of voluntary exercise (1 h/day) and restraint stress (2 h/day) (*n* = 5). *p*‐values were determined using one‐way ANOVA. (B) qRT‐PCR analysis of BDNF expression in the skeletal muscle, heart, hypothalamus and spleen of wild‐type mice exposed to voluntary exercise and restraint stress (*n* = 4). *p*‐values were determined using one‐way ANOVA. (C) Immunofluorescence staining for BDNF (red) in the hypothalamus of C57BL/6 wild‐type mice after voluntary exercise and restraint stress. Scale bar = 50 µm. 3 V, third ventricle. (D) qRT‐PCR analysis of Kif4 mRNA expression in BMDMs treated with rBDNF (0, 5, 25, 50, 100 or 200 ng/mL) (*n* = 3). *p*‐values were determined using two‐sample *t*‐tests. (E) Western blot analysis of TrkB, p‐TrkB, STAT3, p‐STAT3 and Kif4 protein levels in BMDMs treated with rBDNF (100 ng/mL). (F) ChIP‒PCR analysis of p‐STAT3 binding to four Kif4 promoter regions (*n* = 3). *p*‐values were determined using two‐sample *t*‐tests. (G) Western blot analysis of TARM1 protein levels in membrane fractions isolated from BMDMs treated with rBDNF (100 ng/mL). (H) Immunofluorescence staining showing that TARM1 (green) localized to the cell membrane of BMDMs treated with rBDNF (100 ng/mL). Scale bar = 20 µm. (I, J) Flow cytometry gating strategy and quantification of M1 (F4/80^+^CD86^+^) and M2 (F4/80^+^CD206^+^) BMDMs treated with rBDNF (100 ng/ml) (*n* = 5). *p*‐values were determined using two‐sample *t*‐tests. (K) qRT‐PCR analysis of inflammatory and anti‐inflammatory gene expression in BMDMs treated with rBDNF (100 ng/mL) (*n* = 6). *p*‐values were determined using two‐sample *t*‐tests. The data are presented as the means ± SDs.

Next, we investigated how exercise modulated stress‐driven monocyte inflammation. Since BDNF is regulated by exercise and increases peripheral blood plasma BDNF levels,^28^ we hypothesized that the anti‐inflammatory effects of exercise are mediated by plasma BDNF levels and subsequently measured plasma BDNF levels using ELISA. Plasma BDNF levels in exercise‐treated CRS‐exposed mice were higher than those in untreated CRS‐exposed mice (Figure 5A). To better understand the changes in plasma BDNF levels in response to exercise and stress, we isolated the skeletal muscle, heart, hypothalamus and spleen from mice to screen for BDNF tissue sources using qRT‐PCR. We observed higher BDNF expression in the hypothalamus, a brain region closely linked to stress responses, than in the skeletal muscle, heart, or spleen (Figure 5B). Moreover, the immunofluorescence results indicated that the hypothalamic BDNF concentration in exercise‐treated CRS‐exposed mice was significantly higher than that in untreated CRS‐exposed mice (Figure 5C; Figure S6A). Further experiments revealed that recombinant BDNF (rBDNF) treatment downregulated Kif4 expression, with 100 ng/mL rBDNF exerting the most significant effect (Figure 5D). Therefore, we hypothesized that exercise attenuates stress‐driven inflammatory monocytes via the BDNF–Kif4–TARM1 axis.

BDNF activates its receptor TrkB, which suppresses the transcriptional activity of phosphorylated STAT3 (p‐STAT3), thereby attenuating macrophage inflammation.^29^ Further experiments revealed that rBDNF stimulation increased the p‐TrkB/TrkB ratio and decreased the p‐STAT3/STAT3 ratio, accompanied by reduced Kif4 expression (Figure 5E; Figure S6B–D). We determined the mechanism underlying the BDNF‐mediated regulation of Kif4 in monocytes by performing ChIP‒qPCR and found that p‐STAT3 bound to the four promoter regions of the Kif4 mRNA and that rBDNF stimulation significantly inhibited this binding (Figure 5F; Figure S6E). Consistently, rBDNF stimulation decreased the expression of membrane TARM1 and increased the expression of cytoplasmic TARM1 without altering total TARM1 levels (Figure 5G–H; Figure S6F–J). Furthermore, we assessed the effect of BDNF on macrophage inflammation. After rBDNF stimulation, the proportion of M2 macrophages increased in BMDMs, and the expression of anti‐inflammatory genes such as Arg1, CD206, Retnlb and MGL increased (Figure 5I–K). Taken together, these findings indicate that BDNF–TrkB–STAT3 signalling suppresses the Kif4–TARM1 axis, thereby inhibiting macrophage inflammation in vitro.

### 3.6 ANA‐12 blocks the suppressive effect of exercise on stress‐induced inflammatory monocytes

**FIGURE 6:**
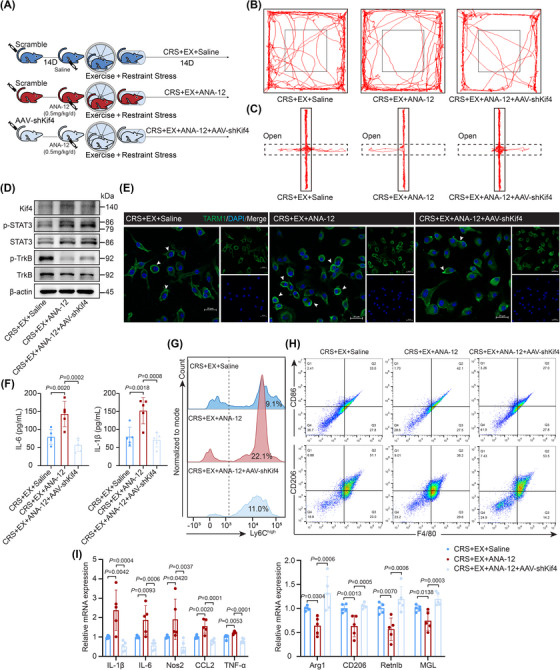
ANA‐12 inhibits the exercise‐mediated suppression of stress‐induced inflammatory monocytes. (A) Experimental schedules. Wild‐type mice received tail vein injections of AAV‐shKif4 or scrambled shRNA as a control. Starting at 14 days post‐injection, the model mice were subjected to daily intraperitoneal injections of ANA‐12 (0.5 mg/kg) or saline concurrently with daily restraint stress (2 h/day) and voluntary wheel running (1 h/day) for 14 days. (B, C) Representative locomotion tracks of model mice in the open‐field test (OFT) and elevated plus maze (EPM) test are shown. (D) Western blot analysis of TrkB, p‐TrkB, STAT3, p‐STAT3 and Kif4 protein levels in BMDMs from model mice. (E) Immunofluorescence staining showing that TARM1 (green) localized to the cell membrane in the BMDMs of the model mice. Scale bar = 20 µm. (F) Plasma IL‐6 and IL‐1β levels measured by ELISA (*n* = 5). (G) Representative flow cytometry plots of CD11b^+^Ly6C^high^ monocytes in peripheral blood. (H) Flow cytometry gating strategy for M1 (F4/80^+^CD86^+^) and M2 (F4/80^+^CD206^+^) BMDMs from model mice. (I) qRT‐PCR analysis of inflammatory and anti‐inflammatory gene expression in BMDMs from model mice (*n *= 5). The data are presented as the means ± SDs. *p*‐values were determined using one‐way ANOVA.

We further investigated whether the BDNF receptor TrkB regulates the Kif4–TARM1 axis in vivo to mediate its anti‐inflammatory effects. To address this, we administered the TrkB inhibitors ANA‐12 and AAV‐shKif4 injection to exercise‐treated CRS‐exposed mice (Figure 6A). The inhibitor ANA‐12 suppressed the expression of TrkB and p‐TrkB in peripheral blood monocytes (Figure 6D; Figure S7C, D), subsequently exacerbating anxiety‐like behaviours (Figure 6B,C; Figure S7A,B), consistent with previous studies.^30^ Moreover, ANA‐12 increased STAT3, p‐STAT3, Kif4 and membrane TARM1 protein levels (Figure 6D,E; Figure S7E–H). In contrast, AAV‐shKif4 treatment decreased Kif4 and membrane TARM1 expression in ANA‐12‐injected exercise‐treated CRS‐exposed mice (Figure 6D,E; Figure S7G,H).

Consistent with previous data, we observed that the inhibitor ANA‐12 increased plasma IL‐6 and IL‐1β levels, as well as the proportion of CD11b^+^Ly6C^high^ monocytes, whereas AAV‐shKif4 treatment reversed these proinflammatory responses (Figure 6F,G; Figure S7I). Similarly, ANA‐12 increased M1 macrophage polarization and the expression of M1 macrophage markers (IL‐1β, IL‐6, Nos2, CCL2 and TNF‐α), while AAV‐shKif4 reversed these changes (Figure 6H,I; Figure S7J). Collectively, these results highlight the role of the BDNF–Kif4–TARM1 axis in the modulation of stress‐driven inflammatory monocytes by exercise and reveal that exercise regulates neuroimmune crosstalk.

### 3.7 Exercise mitigates stress‐induced vascular inflammation via the BDNF–Kif4–TARM1 axis in ApoE^−/−^ mice

Based on the above findings, we sought to determine whether exercise mitigates stress‐induced atherosclerotic vascular inflammation involving the BDNF–Kif4–TARM1 axis. We tested the hypothesis that chronic stress promotes plaque progression and destabilization through mechanisms involving inflammatory monocytes. We exposed atherosclerosis‐prone mice (ApoE^−/−^ mice fed an HFD for 8 weeks) to CRS (Figure S8A), which led to anxiety‐like behaviours (Figure S8B–E). Based on the data obtained from stress‐exposed ApoE^−/−^ mice, the expression of Kif4 was higher in peripheral blood monocytes and aortic plaque macrophages than in non‐stressed ApoE^−/−^ mice (Figure S8F–H). Moreover, ELISA of peripheral blood plasma revealed that stress exposure decreased BDNF levels but increased IL‐6 and IL‐1β levels (Figure S8I,J). Flow cytometry further confirmed a surge in the proportion of pro‐inflammatory CD11b^+^Ly6C^high^ monocytes and M1 macrophages in stress‐exposed ApoE^−/−^ mice (Figure S8K–N). Notably, stress exposure increased atherosclerotic vascular inflammation via proinflammatory monocytes, thereby increasing aortic plaque area and plaque destabilization (Figure S8O–U).

**FIGURE 7:**
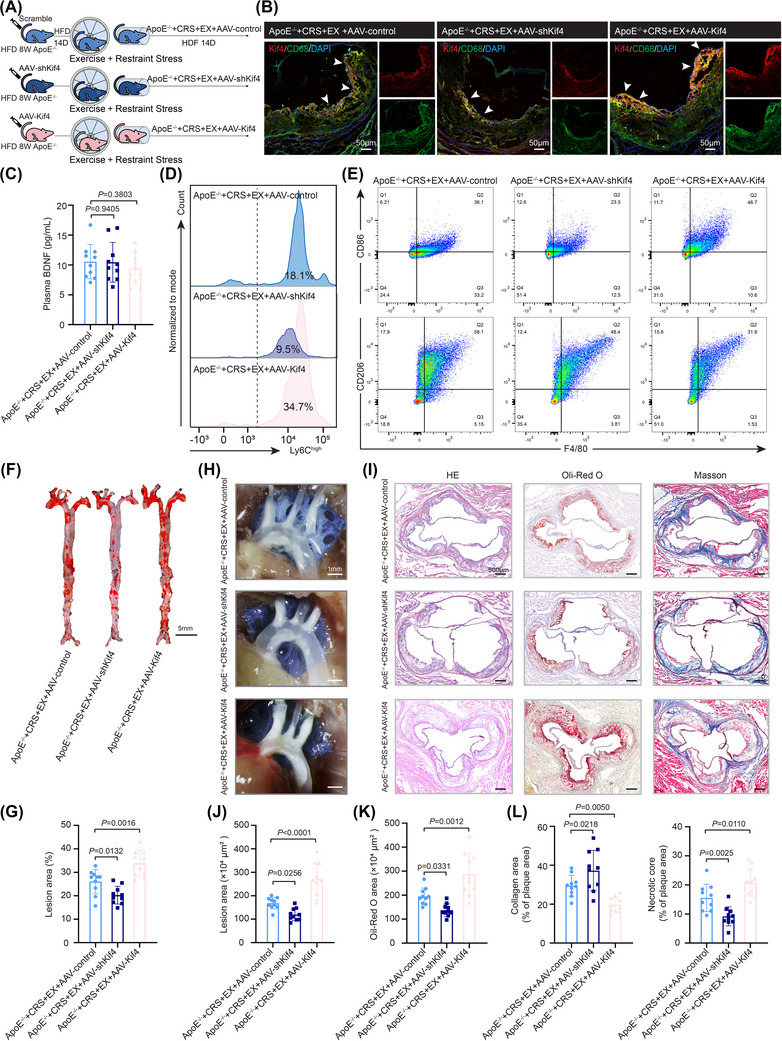
The BDNF–Kif4–TARM1 axis is involved in the exercise‐mediated reduction in vascular inflammation and plaque stabilization in model mice. (A) Experimental schedules. ApoE^−/−^ mice were fed an HFD for 12 weeks to establish an atherosclerosis model. At week 9 of the HFD, the mice were intravenously injected with AAV‐shKif4 (Kif4 knockdown), AAV‐Kif4 (Kif4 overexpression), or scrambled control AAV. Beginning at week 11 of the HFD, the mice were subjected to daily restraint stress (2 h/day) and voluntary exercise (1 h/day) for 2 weeks. (B) Immunofluorescence staining of atherosclerotic plaques showing CD68^+^ macrophages (green), Kif4 (red) and DAPI (blue). Scale bar = 50 µm. (C) Plasma BDNF levels in atherosclerotic mice measured by ELISA (*n* = 10). (D) Representative flow cytometry plots of CD11b^+^Ly6C^high^ monocytes in peripheral blood. (E) Flow cytometry gating strategy for M1 (F4/80^+^CD86^+^) and M2 (F4/80^+^CD206^+^) BMDMs from atherosclerotic mice. (F) Representative images of Oil Red O staining of total aortic plaques. Scale bar = 5 mm. (G) Analysis of Oil Red O staining to determine the total aortic plaque area (*n* = 10). (H) Representative images of aortic atherosclerotic plaques. Scale bar = 1 mm. (I) Representative images showing the histopathology of aortic sinus plaques. Scale bar = 500 µm. (J, K) Plaque size assessments via HE staining and Oil Red O staining (*n* = 10). (L) Necrotic core and collagen fibre area in aortic sinus plaques measured by Masson's trichrome staining (*n* = 10). The data are presented as the means ± SDs. *p*‐values were determined using one‐way ANOVA.

Next, we investigated whether exercise mitigated stress‐induced vascular inflammation through the kinesin Kif4, thereby modulating plaque characteristics in ApoE^−/−^ mice. In exercise‐treated CRS‐exposed ApoE^−/−^ mice injected with AAV‐shKif4 or AAV‐Kif4 (Figure 7A), the behavioural phenotypes did not differ under the same stress‐modelling conditions (Figure S9A–D). Kif4 expression was significantly lower in the aortic root plaques of AAV‐shKif4‐treated ApoE^−/−^ mice than in control mice, and BDNF levels did not differ under the same exercise conditions (Figure 7B,C; Figure S9E). Consistent with the findings from WT mice, AAV‐shKif4 treatment significantly reduced the proportion of proinflammatory CD11b^+^Ly6C^high^ monocytes and M1 macrophages, as well as the plasma levels of IL‐6 and IL‐1β in ApoE^−/−^ mice (Figure 7D,E; Figure S7F–H). Importantly, we found that Kif4 knockdown decreased the plaque area in the total aorta and aortic arch of AAV‐shKif4‐treated ApoE^−/−^ mice (Figure 7F–H). Consistent with these findings, analysis of the aortic root revealed a decreased plaque area and necrotic core, accompanied by increased collagen deposition (Figure 7I–L). Taken together, our data indicate that exercise mitigates stress‐induced vascular inflammation, thereby promoting plaque stabilization through suppression of inflammatory monocytes via the BDNF–Kif4–TARM1 axis.

### 3.8 Regular exercise attenuates psychological stress‐induced inflammation in patients with CAD via the BDNF–Kif4–TARM1 axis

**FIGURE 8:**
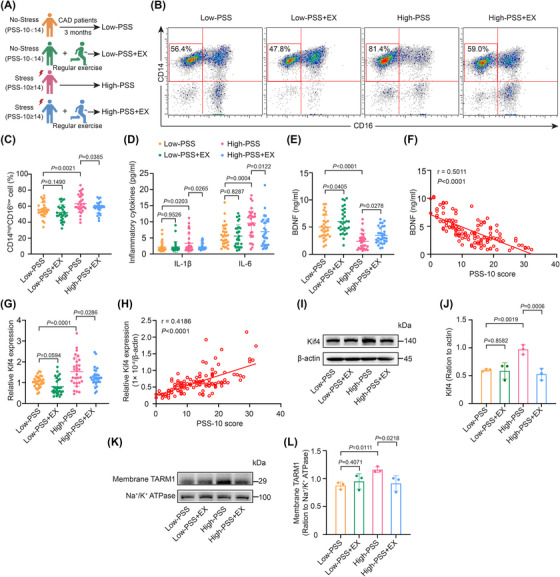
The anti‐inflammatory effect of regular exercise on psychological stress in CAD patients involves the BDNF–Kif4–TARM1 axis. (A) Experimental schematic. CAD patients enrolled at the 3‐month outpatient follow‐up visit were categorized into four groups (*n* = 30 patients/group) on the basis of the following parameters: PSS‐10 scores (low vs. high) and regular exercise habits (≥3–5 aerobic sessions/week). (B, C) Representative flow cytometry plots and quantification of CD14^high^CD16^low^ monocytes in the peripheral blood of CAD patients (*n* = 30). (D) Plasma IL‐6 and IL‐1β levels quantified by ELISA (*n* = 30). (E) Plasma BDNF levels in CAD patients measured by ELISA (*n* = 30). (F) Analysis of the correlation between plasma BDNF levels and PSS‐10 scores (*n* = 120). (G) qRT‐PCR analysis of Kif4 expression in peripheral blood monocytes from CAD patients (*n* = 30). (H) Analysis of the correlation between Kif4 expression levels and PSS‐10 scores (*n* = 120). (I, J) Western blot analysis of Kif4 protein levels in peripheral blood monocytes from CAD patients (*n* = 3). (K, L) Western blot analysis of TARM1 protein levels in membrane fractions isolated from the peripheral blood monocytes of patients with CAD (*n* = 3). The data are presented as the means ± SDs. *p*‐values were determined using one‐way ANOVA.

To generalize these rodent data to humans, we recruited patients with CAD at the 3‐month post‐discharge follow‐up and grouped them based on their physical activity levels and PSS‐10 questionnaire results (Figure 8A). Demographic and clinical characteristics, including age, sex and medical history (hypertension, diabetes, previous stroke and current smoker), did not differ significantly between the groups (Table S1). Notably, white blood cell counts were significantly different between groups, whereas metabolic and cardiovascular indicators [body mass index (BMI), left ventricular ejection fraction (LVEF), creatinine levels, HbA1c levels and lipid profiles] were not significantly different (Table S1). Furthermore, we observed that the proportion of inflammatory CD14^high^CD16^low^ monocytes and the levels of inflammatory cytokines were elevated in patients with high stress levels, whereas regular exercise attenuated these responses (Figure 8B–D). Moreover, regular exercise downregulated Kif4 and TARM1 expression in monocytes and increased plasma BDNF levels in patients with CAD with high stress levels (Figure 8E, G, I–L). We also found that stress severity was negatively correlated with plasma BDNF levels and positively correlated with peripheral blood monocytic Kif4 expression (Figure 8F,H). Collectively, these data indicate that regular exercise exerts anti‐inflammatory effects in stressed individuals via the BDNF–Kif4–TARM1 axis.

## 4 DISCUSSION

Psychosocial stress, a nonclassical risk factor, plays a critical role in the pathogenesis of atherosclerosis.^31^ Unlike traditional risk factors, such as hypercholesterolemia, smoking and ageing, which have been extensively studied,^32^, ^33^, ^34^ psychological stress remains understudied; therefore, the underlying mechanisms and potential intervention strategies are of significant interest. Chronic stress‐induced inflammation is a key driver of atherosclerosis.^35^ Exercise intervention has significant effects on many diseases, including atherosclerosis.^36^ The benefits of physical exercise for atherosclerosis associated with reduced inflammation^37^; however, little is known about the mechanisms mediating the effects of stress‐induced vascular inflammation. Here, we report that exercise alleviates stress‐induced neuroimmune crosstalk, thereby reducing vascular inflammation. Furthermore, our data confirmed that exercise partially attenuates vascular inflammation through a reduction in the proportion of CD11b^+^Ly6C^high^ monocytes in mice and CD14^high^CD16^low^ monocytes in patients with CAD, a process mediated by the BDNF–Kif4–TARM1 axis.

The anti‐inflammatory effects of exercise have previously been linked to reduced levels of inflammatory monocytes and cytokines in the blood.^14^ Monocytes are the key to sustaining systemic inflammation to accelerate inflammatory diseases (e.g., atherosclerosis), while psychosocial stress remodels their chromatin and transcriptome, skewing monocytes towards a hyperinflammatory state in both mice and humans.^38^ To our knowledge, this study is the first to explore the role of exercise in stress‐induced inflammatory monocyte reprogramming. We demonstrate that exercise reduces proinflammatory monocytes in CRS‐exposed mice and patients with CAD with high PSS scores. Moreover, exercise decreased circulating IL‐1β and IL‐6 under stress conditions. These findings underscore the need to further elucidate the molecular mechanisms underlying the beneficial effects of exercise on stress‐induced vascular inflammation, which may inform new therapeutic strategies for preventing and treating psychogenic cardiovascular inflammation and related diseases.

Through RNA transcriptional analysis, we identified Kif4 as a key mediator linking exercise to stress‐induced monocyte inflammation. Kif4, a member of the kinesin superfamily of proteins (KIFs), controls cellular morphogenesis and function via intracellular transport, tightly regulating the direction, destination and velocity of critical functional molecules.^39^, ^40^ In this context, we hypothesized that external stimuli, such as exercise and stress, may alter the monocyte microenvironment, necessitating regulated Kif4 expression to maintain cellular adaptive regulation. Evidence indicates that both in highly differentiated and polarized cells, such as neurons and glia, and during the invasion of the hepatitis virus into liver cells, an increased number of KIFs is required to support their sophisticated functional adaptations.^24^, ^39^ Here, we and others have shown that Kif4 regulates inflammatory responses in monocytes and macrophages.^22^, ^41^ Our study further emphasizes the role of Kif4 in exercise‐regulated neuroimmune responses, as validated via Kif4 knockout cell lines and AAV‐mediated Kif4 knockdown or overexpression. Our data from mouse intervention models and observations of patients with CAD indicate that targeting the motor protein Kif4 may represent a novel therapeutic strategy for reducing stress‐induced inflammatory monocytes, although other mechanisms are likely involved. Critically, advanced studies on the functions of KIFs in health and disease, a field where current knowledge is both limited and inadequately prioritized, are needed.

Because Kif4 transports protein complexes and membranous organelles in a microtubule‐dependent manner,^42^ we performed CoIP‒MS and protein mass spectrometry sequencing to identify the downstream targets of the motor protein Kif4. Through these approaches, we found that plasma membrane TARM1, a key Kif4‐downstream effector, regulates its cellular distribution in macrophages upon Kif4 knockdown or overexpression. Immunofluorescence staining confirmed that Kif4 governs TARM1 distribution via microtubule‐based transport. As a leukocyte immunoglobulin‐like receptor (LIR) family member, TARM1 stimulates macrophage proinflammatory responses and increases IL‐6/TNF‐α production; targeting membrane‐surface TARM1 with the TARM1‐Fc fusion protein inhibits these effects, as shown in our study and other previous reports.^43^ Consistent with a recent report,^44^ TARM1 activates macrophage inflammatory responses; conversely, macrophages counteract this by degrading plasma membrane TARM1 to suppress renal inflammation and protect against acute kidney injury. Notably, plasma membrane TARM1 not only activates the proinflammatory response of immune cells themselves but may also costimulate inflammatory responses in other related cell types.^25^ Given the role of monocyte‒endothelial cell interactions in the development of vascular inflammation,^26^ we further found that TARM1‐Fc‐treated THP‐1 cells improved HUVEC migration and reduced HUVEC adhesion, oxidative stress and mitochondrial membrane potential damage in a coculture system of THP‐1 cells and HUVECs. Consistent with the findings of Symes et al.,^45^ chronic stress elevates the levels of inflammatory factors and VCAM‐1, exacerbating the progression of atherosclerosis. Therefore, our findings indicate that Kif4 affects stress‐induced macrophage proinflammatory responses, partly through microtubule‐dependent TARM1 transport. Notably, surface TARM1 in monocytes and macrophages may contribute to endothelial cell dysfunction, suggesting a role in vascular inflammation.

Substantial evidence has demonstrated that neuroimmune crosstalk involves multiple pathways.^46^ The mental health benefits of physical exercise are tied to increased brain BDNF levels.^47^ This elevation further increases plasma BDNF levels^17^; notably, 70–80% of blood‐circulating BDNF is derived from the brain.^48^ In addition, BDNF significantly affects the nervous, immune and cardiovascular systems.^49^ Our data, in line with these studies, reveal that exercise significantly increases BDNF expression in the hypothalamus of CRS‐exposed mice and that this effect also extends to plasma BDNF levels. Previous reports suggest that the integration of neuroimmune signals by the hypothalamus underpins its significant role and makes it a key area of interest in the chronic stress response.^50^ Moreover, exercise increases plasma BDNF levels in patients with CAD with high PSS scores, and increased stress severity is negatively correlated with plasma BDNF levels in this population. These findings are consistent with studies showing that (1) increased academic stress is associated with decreased plasma BDNF levels in Chilean college students and (2) patient health questionnaire‐9 (PHQ‐9) scores are inversely correlated with serum BDNF levels in patients with CAD who have comorbid depressive symptoms.^51^, ^52^ Building on existing evidence, we propose that BDNF regulates neuroimmune crosstalk and functions as a critical signalling mediator bridging the brain–heart axis. Specifically, BDNF‐dependent signalling predominantly activates the receptor TrkB.^53^ Therefore, we stimulated BMDMs with rBDNF and injected ANA‐12 (a selective TrkB inhibitor) into exercise‐treated CRS‐exposed mice to test this hypothesis. We subsequently revealed that BDNF–TrkB–STAT3 signalling suppresses the Kif4–TARM1 axis, effectively inhibiting macrophage inflammation in vitro. We also found that ANA‐12 significantly impaired the BDNF‐mediated anti‐inflammatory activity in exercise‐treated CRS‐exposed mice. These findings partly align with a study demonstrating that environmental eustress promotes hypothalamic BDNF expression, which further affects plasma BDNF levels and acts on cardiac macrophages.^54^ Furthermore, we observed that exercise mitigates stress‐induced vascular inflammation via the BDNF–Kif4–TARM1 axis in both ApoE^−/−^ mice and patients with CAD. Collectively, these results indicate that the neuroimmune mechanisms through which exercise alleviates stress‐induced vascular inflammation involve the upregulation of hypothalamic BDNF expression, which elevates plasma BDNF levels, thereby inhibiting systemic monocyte inflammation via the Kif4–TARM1 axis.

This study has some limitations that warrant consideration. Given the essential functions of Kif4 in fundamental cellular processes (e.g., mitosis), Kif4 knockout mice exhibit profound embryonic lethality, precluding in vivo validation using this model. The proinflammatory mechanism of monocyte surface TARM1, particularly its effects on endothelial cells and other cells, requires further study. To generalize these rodent data to humans, we enrolled four groups of patients with CAD. However, sample size limitations introduced potential confounding factors. Future studies on the anti‐inflammatory efficacy of exercise in the treatment of psychogenic diseases in larger cohorts are needed. Despite these limitations, our study provides important mechanistic evidence of how exercise alleviates stress‐induced vascular inflammation.

In conclusion, our study provides new insights into exercise‐regulated neuroimmune mechanisms partly mediated by the BDNF–Kif4–TARM1 axis. Exercise and exercise‐induced factors, including BDNF, represent potential therapeutic strategies for patients with psychogenic CAD.

## AUTHOR CONTRIBUTIONS

**Xianghui Zheng, Peiyao Wang** and **Zhou Guo**: Writing—review and editing, writing—original draft, methodology, investigation, formal analysis, data curation and conceptualization. **Yunqi Li** and **Yuxuan Liu**: Writing—review and editing, methodology and conceptualization. **Qi Liu, Baitao Wang, Lizhi Zheng, Cien Li, Shuhong Liu, Shiyu Wanga** and **Xiaojun Wu**: Writing—review and editing and methodology. **Huiyu Wang** and **Xinyu Hou**: Writing—review and editing and data curation. **Yong Sun**: Writing—review and editing and supervision. **Bo Yu**: Writing—review and editing and resources. **Yang Zheng**: Writing—review and editing, methodology, investigation, funding acquisition, formal analysis, data curation and conceptualization. **Jian Wu**: Project administration, funding acquisition and conceptualization.

## ETHICS STATEMENT

The animal and participant protocols were approved by the Experimental Animal Welfare and Ethics Committee of the Second Affiliated Hospital of Harbin Medical University, Harbin city (No. YJSDW2023‐250).

## CONFLICT OF INTEREST STATEMENT

The authors declare no conflict of interest.

## Supporting information



Supporting Information

Supporting Information

Supporting Information

## Data Availability

The data underlying this article are available in the article and in its online supplementary material. All the data that support the findings of this study are available from the corresponding author upon reasonable request.
