# Supplementary material for: Physical exercise mitigates chronic psychological stress‐induced vascular inflammation via the BDNF–Kif4–TARM1 axis
Source: Clin Transl Med. 2026 Apr 20;16(4):e70674. doi: 10.1002/ctm2.70674 (PMC13096691; doi:10.1002/ctm2.70674)
Supplement: Supplementary file 1 — Supporting Information [file CTM2-16-e70674-s002.docx]

**Supplementary Figures**

**
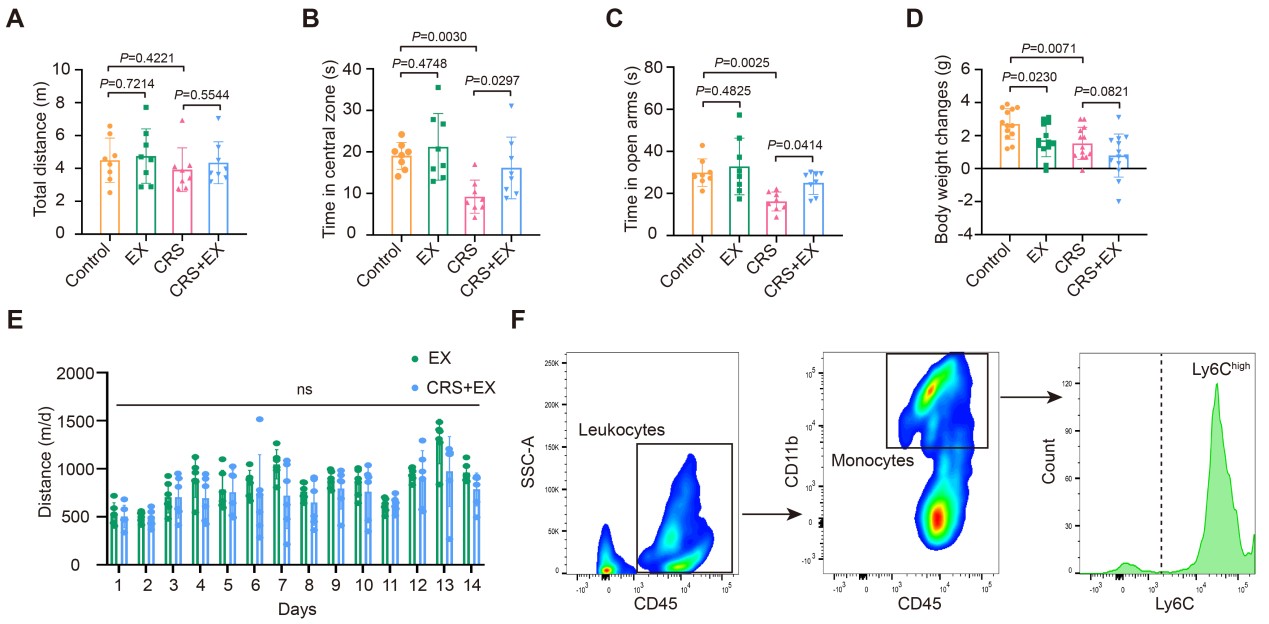
**

**Figure S1. Voluntary exercise reduces stress-induced anxiety-like behaviors and systemic inflammation. (A)** Total distance traveled in the open field test (n = 8). *P* values were determined by one-way ANOVA. **(B)** Duration in the central area of the open field (n = 8). *P* values were determined using one-way ANOVA. **(C)** Time spent in the open arms of the elevated plus maze (n = 8). *P* values were determined by one-way ANOVA. **(D)** Changes in body weight 14 days after modeling (n = 13). *P* values were determined using one-way ANOVA. **(E)** Daily running wheel distance of mice in the EX and CRS+EX groups (n = 6). *P* values were determined using two-sample t tests. **(F)** Flow cytometry gating strategy for peripheral blood monocytes. The data are presented as the means ± SDs.

**
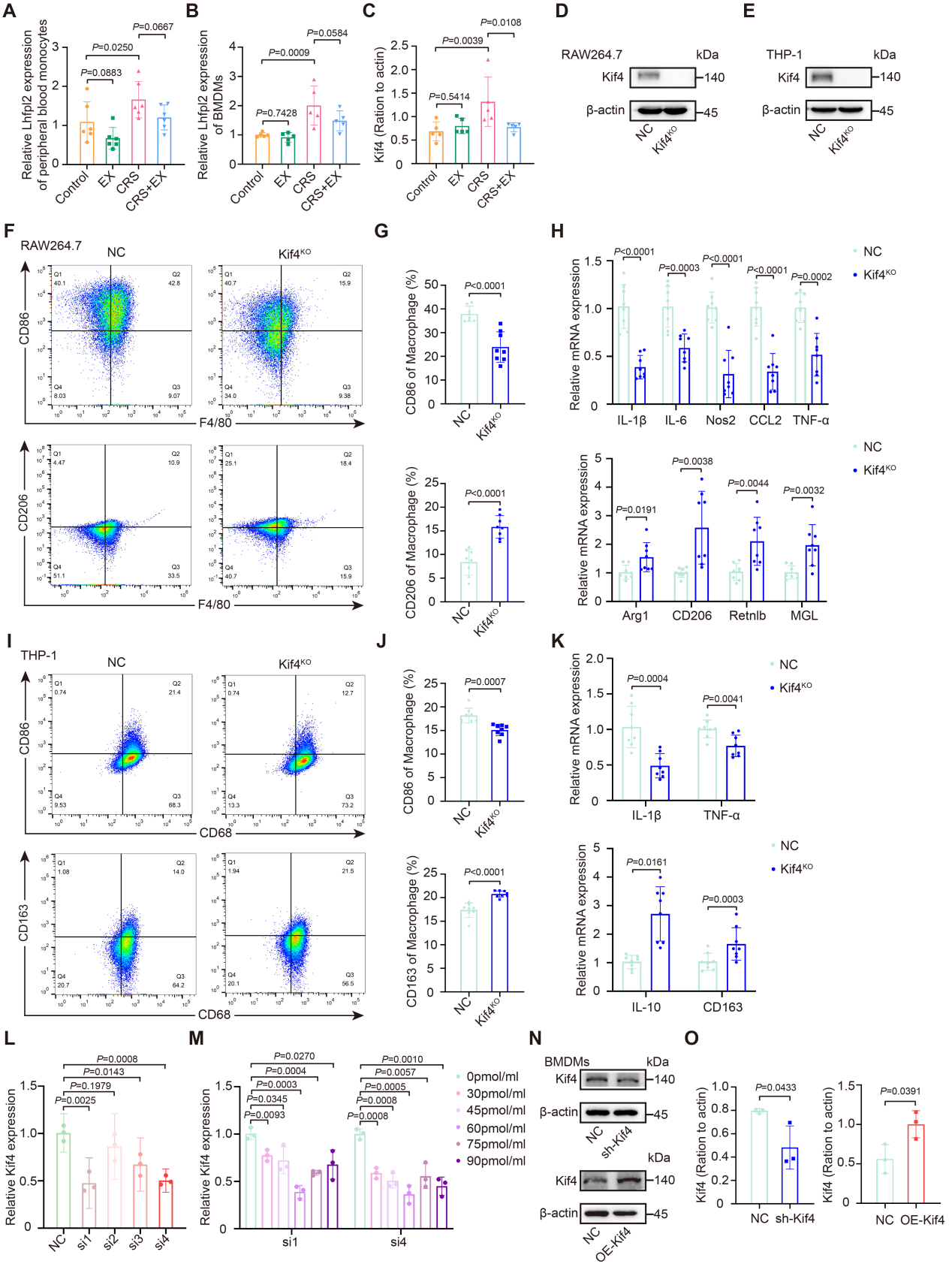
**

**Figure S2. Targeting Kif4 enables the exercise-mediated attenuation of stress-induced inflammatory monocytes. (A, B)** qRT-PCR analysis of Lhfpl2 expression in peripheral blood monocytes (n = 6) and BMDMs (n = 5). *P* values were determined using one-way ANOVA. **(C)** Quantification of relative Kif4 protein expression (n = 5). *P* values were determined using one-way ANOVA. **(D, E)** Western blot analysis of Kif4 expression in Kif4 knockout RAW264.7 cells and Kif4 knockout THP-1 cells. **(F, G)** Flow cytometry analysis: gating strategy and quantification of RAW264.7 or Kif4 knockout RAW264.7 cells (n = 8). *P* values were determined using two-sample t tests. **(H)** qRT-PCR analysis of inflammatory and anti-inflammatory gene expression in RAW264.7 or Kif4 knockout RAW264.7 cells (n = 8). *P* values were determined using two-sample t tests. **(I, J)** Flow cytometry analysis: gating strategy and quantification of THP-1 or Kif4 knockout THP-1 cells (n = 8). *P* values were determined using two-sample t tests. **(K)** qRT-PCR analysis of inflammatory and anti-inflammatory gene expression in THP-1 or Kif4 knockout THP-1 cells (n = 8). *P* values were determined using two-sample t tests. **(L)** qRT-PCR analysis of Kif4 expression in BMDMs after transfection with four distinct Kif4-siRNAs (n = 3). *P* values were determined using two-sample t tests. **(M)** qRT-PCR analysis of Kif4 expression in BMDMs after treatment with graded concentrations of siKif4-1 (si1) and siKif4-4 (si4) (n = 3). *P* values were determined using two-sample t tests. **(N, O)** Western blot analysis of Kif4 expression in BMDMs after transfection with OE-Kif4 or sh-Kif4 plasmids (n = 3). *P* values were determined using two-sample t tests; the data are presented as the means ± SDs.

**
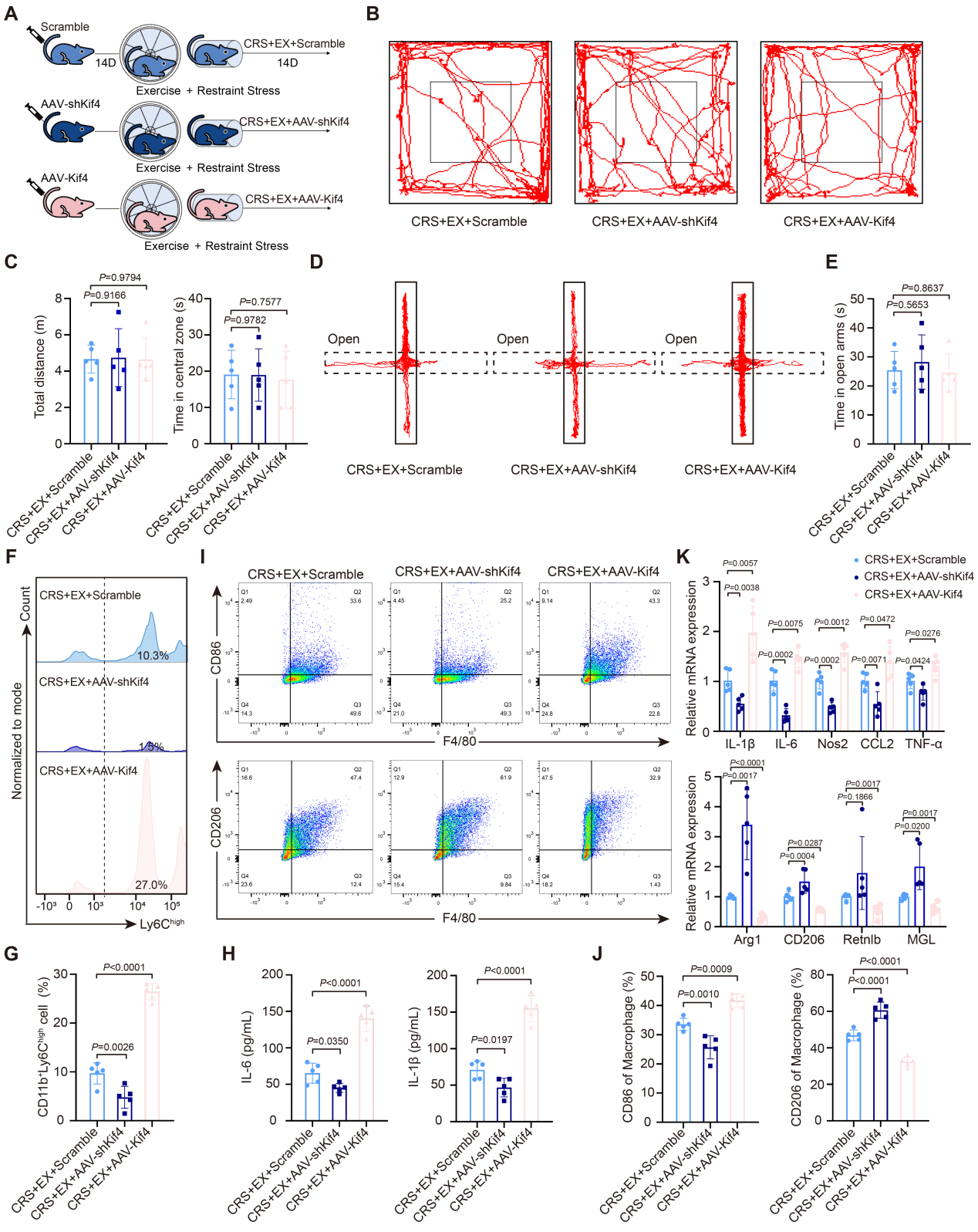
**

**Figure S3. Kif4 suppression is critical for attenuating inflammatory responses in stress-induced monocytes and has effects similar to those of exercise. (A)** Experimental schematic. Wild-type mice received tail vein injections of scrambled plasmid, AAV-shKif4, or AAV-Kif4. Fourteen days after the injection, mice in all groups underwent daily restraint stress (2 h/day) and performed voluntary wheel running (1 h/day) for 14 consecutive days. **(B,** **C)** Representative locomotion tracks in the open field test (OFT) and total distance traveled and time spent in the center zone of the open field (n = 5). **(D, E)** Representative locomotion tracks in the elevated plus maze (EPM) test and time spent in the open arms of the EPM (n = 5). **(F, G)** Representative flow cytometry plots and quantification of CD11b^+^Ly6C^high^ monocytes in peripheral blood (n = 5). **(H)** Plasma IL-6 and IL-1β levels measured by ELISA (n = 5). **(I, J)** Flow cytometry analysis: gating strategy and quantification of BMDMs (n = 5). **(K)** qRT-PCR analysis of inflammatory and anti-inflammatory gene expression in BMDMs (n = 5). The data are presented as the means ± SDs. *P* values were determined using one-way ANOVA.

**
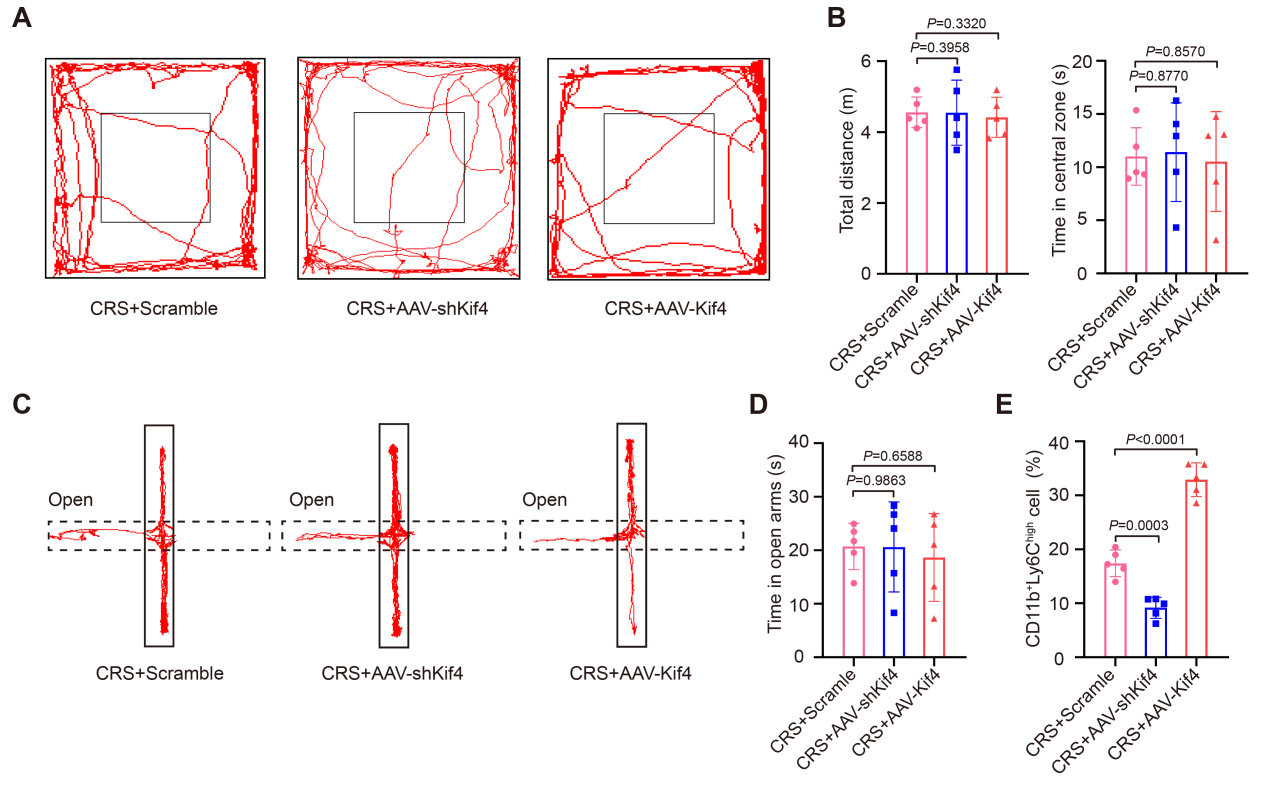
**

**Figure S4. Targeting Kif4 enables the exercise-mediated attenuation of stress-induced inflammatory monocytes. (A)** Representative locomotion tracks of mice in the open field test (OFT). **(B)** Total distance traveled and time spent in the center zone of the open field by mice in the different groups (n = 5). **(C, D)** Representative locomotion tracks in the elevated plus maze (EPM) test and time spent in the open arms of the EPM (n = 5). **(E)** Quantification of CD11b^+^Ly6C^high^ monocytes by flow cytometry (n = 5). The data are presented as the means ± SDs. *P* values were determined using one-way ANOVA.

**
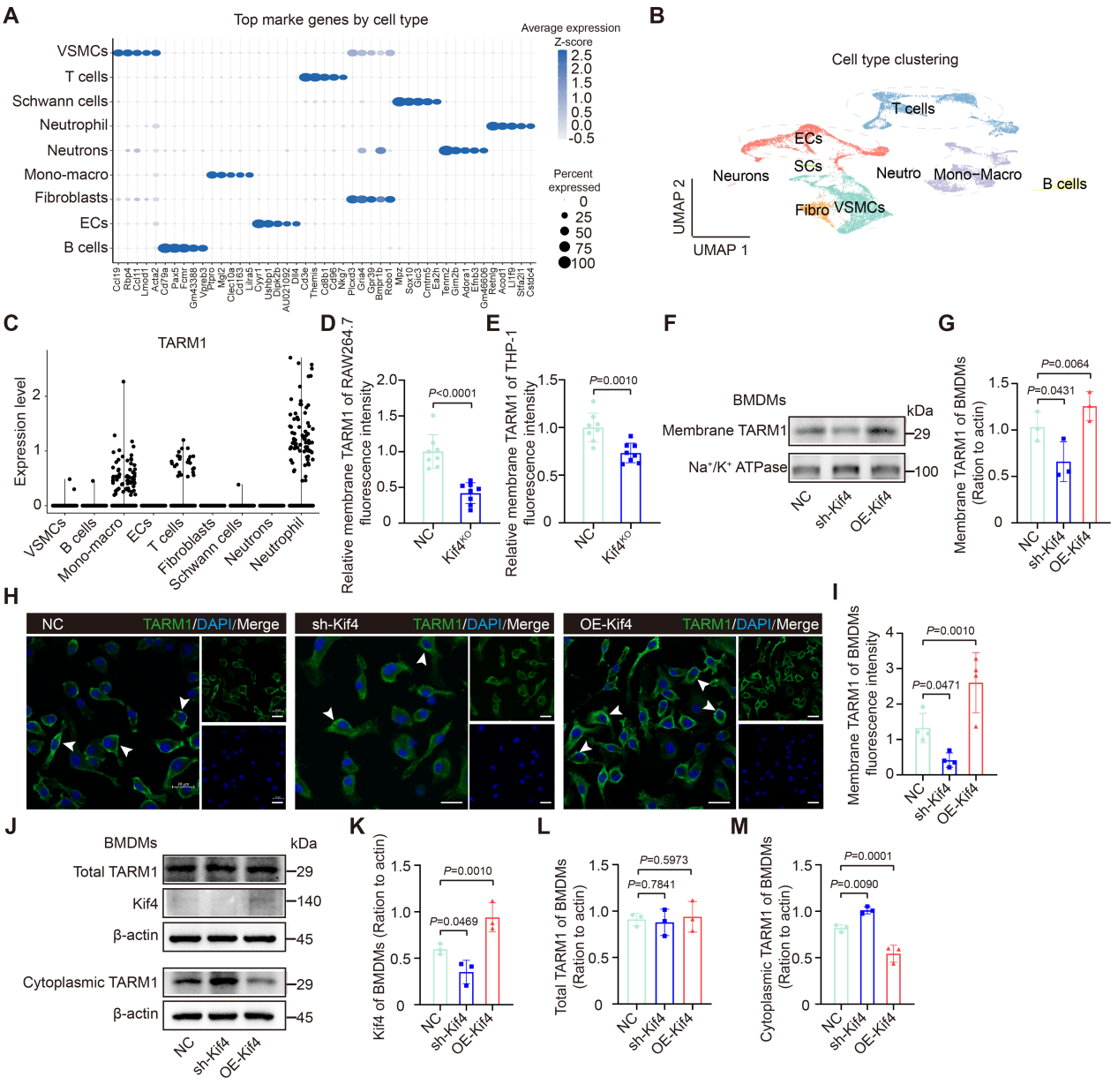
**

**Figure S5. Kif4 promotes the microtubule-dependent delivery of TARM1 to the plasma membrane, activating macrophage inflammation. (A)** Dot plot analysis of the top 5 genes expressed per cluster to identify cell types. VSMCs, vascular smooth muscle cells; Mono-macro, monocytes and macrophages; ECs, endothelial cells. **(B)** UMAP plot of aortic cells from samples, colored by major cell types. **(C)** Analysis of TARM1 expression in major cell types. **(D)** Quantification of membrane-localized TARM1 relative immunofluorescence intensity in RAW264.7 or Kif4 knockout RAW264.7 cells (n = 8). **(E)** Quantification of membrane-localized TARM1 relative immunofluorescence intensity in THP-1 or Kif4 knockout THP-1 cells (n = 8). **(F, G)** Western blot analysis of TARM1 protein levels in membrane fractions isolated from BMDMs transfected with OE-Kif4 or sh-Kif4 plasmids (n = 3). **(H, I)** Immunofluorescence staining showing that TARM1 (green) localized to the cell membrane in BMDMs transfected with OE-Kif4 or sh-Kif4 plasmids (n = 4). Scale bar = 20 µm. **(J-M)** Western blot analysis of TARM1, Kif4, and cytoplasmic TARM1 protein levels in BMDMs transfected with OE-Kif4 or sh-Kif4 plasmids (n = 3). The data are presented as the means ± SDs. *P* values were determined using two-sample t tests or one-way ANOVA.


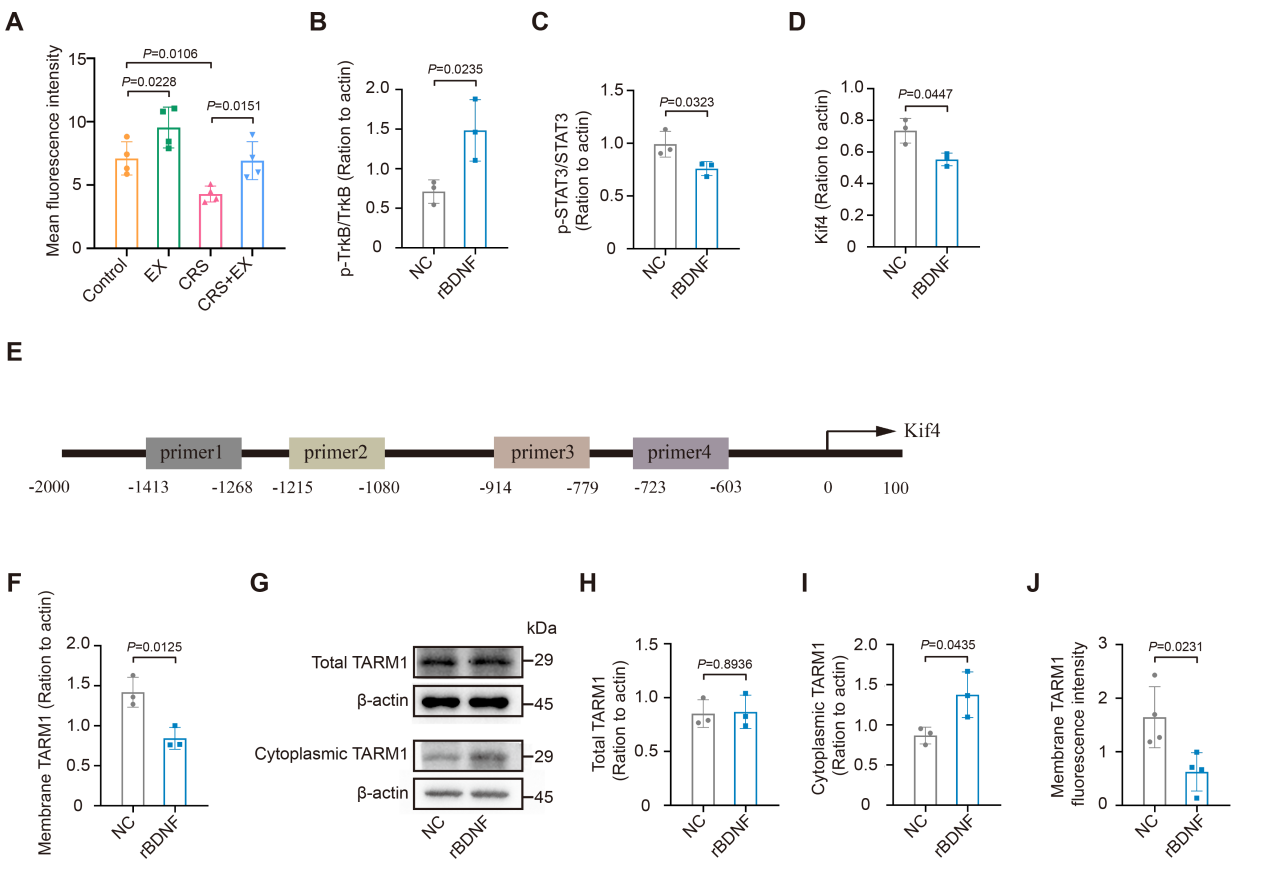


**Figure S6. BDNF–TrkB–STAT3 signaling suppresses the Kif4–TARM1 axis to inhibit macrophage inflammation. (A)** Quantification of BDNF immunofluorescence intensity in the hypothalamus of C57BL/6 wild-type mice following voluntary exercise and restraint stress (n = 4). *P* values were determined using one-way ANOVA. **(B–D)** Quantification of p-TrkB/TrkB, p-STAT3/STAT3, and Kif4 protein levels in BMDMs treated with rBDNF (100 ng/ml) (n = 3). *P* values were determined using two-sample t tests. **(E)** Schematic of the four designed Kif4 promoter regions. **(F)** Quantification of TARM1 protein levels in membrane fractions from BMDMs treated with rBDNF (100 ng/ml) (n = 3). *P* values were determined using two-sample t tests. **(G–I)** Western blot analysis of TARM1 and cytoplasmic TARM1 protein levels in BMDMs treated with rBDNF (100 ng/ml) (n = 3). *P* values were determined using two-sample t tests. J) Quantification of membrane-localized TARM1 immunofluorescence intensity in BMDMs treated with rBDNF (100 ng/ml) (n = 4). *P* values were determined using two-sample t tests. The data are presented as the means ± SDs.

**
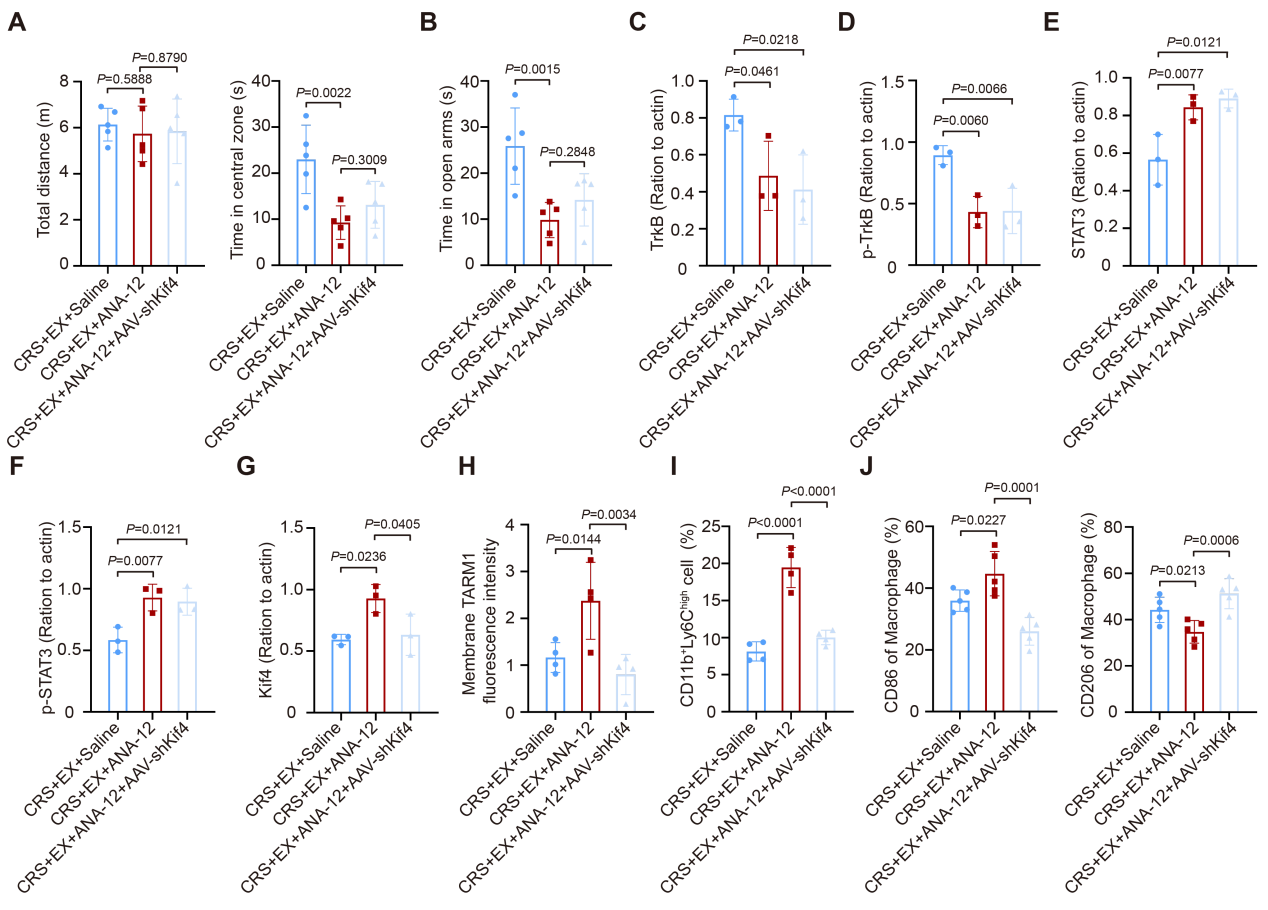
**

**Figure S7. ANA-12 inhibits the exercise-mediated suppression of stress-induced inflammatory monocytes. (A)** Total distance traveled and time spent in the center zone in the open field (n = 5). **(B)** Time spent in the open arms of the elevated plus maze (n = 5). **(C–G)** Quantification of TrkB, p-TrkB, STAT3, p-STAT3, and Kif4 protein levels in BMDMs form model mice (n = 3). **(H)** Quantification of membrane-localized TARM1 immunofluorescence intensity in BMDMs from model mice (n = 4). **(I)** Quantification of CD11b^+^Ly6C^high^ monocytes in the peripheral blood of model mice (n = 4). **(J)** Quantification of M1 (F4/80^+^CD86^+^) and M2 (F4/80^+^CD206^+^) BMDMs from model mice (n = 5). The data are presented as the means ± SDs. *P* values were determined using one-way ANOVA.

**
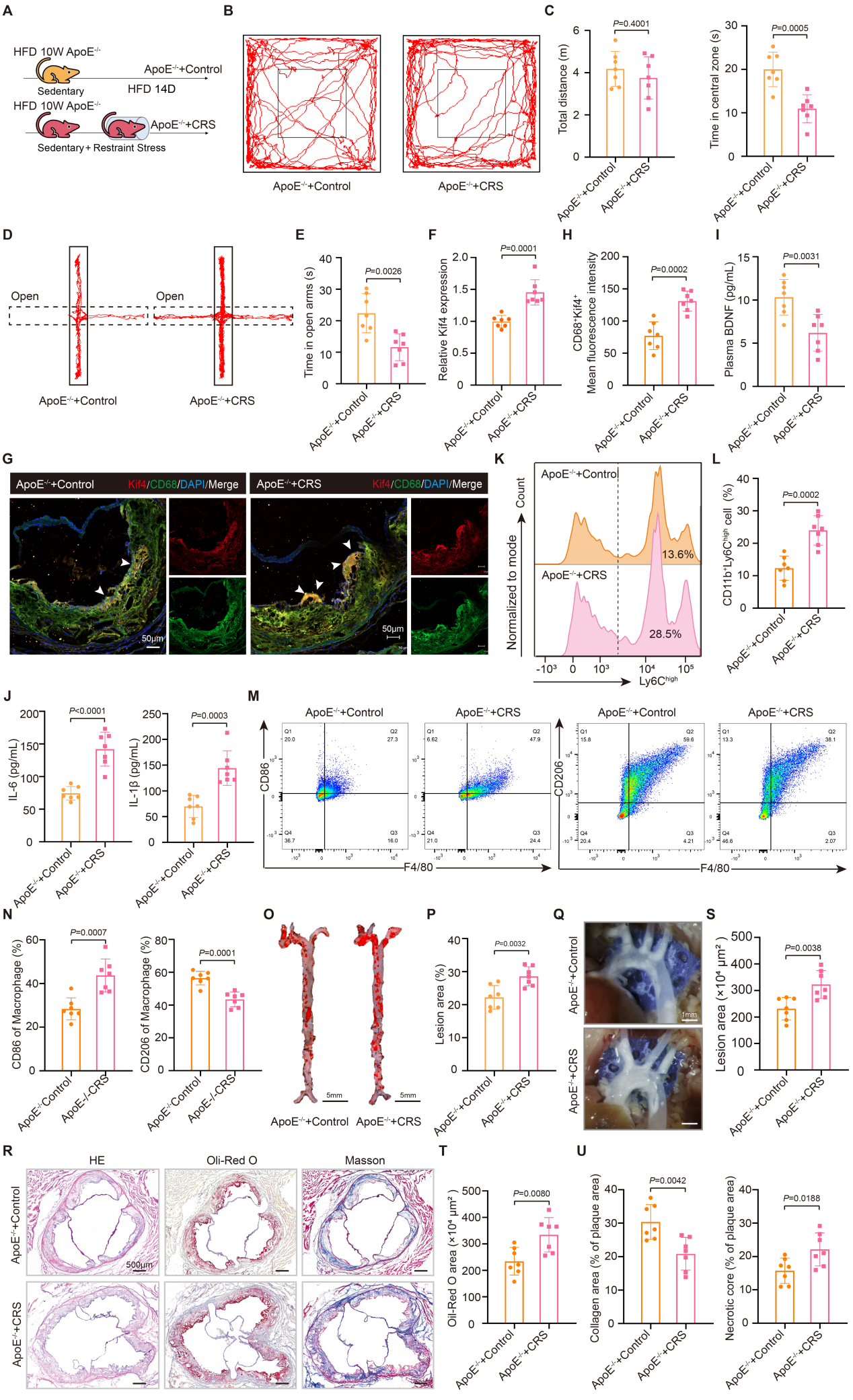
**

**Figure S8. Stress promotes vascular inflammation via proinflammatory monocytes, accelerating atherosclerosis and plaque destabilization. (A)** Experimental schedules. ApoE^-/-^ mice received a high-fat diet (HFD) for 12 weeks (atherosclerosis model) with 2 hours of concurrent daily restraint stress from weeks 11–12 (CRS model). **(B)** Representative locomotion tracks of model mice in the open field test (OFT). **(C)** Total distance traveled and time spent in the center zone of the open field by model mice (n = 7). **(D, E)** Representative locomotion tracks in the elevated plus maze (EPM) test and time spent in the open arms of the EPM by model mice (n = 7). **(F)** qRT-PCR analysis of Kif4 expression in peripheral blood monocytes from model mice (n = 7). **(G)** Immunofluorescence staining of atherosclerotic plaques showing CD68^+^ macrophages (green), Kif4 (red), and DAPI (blue). Scale bar = 50 μm. **(H)** Quantification of the CD86^+^Kif4^+^ immunofluorescence intensity in atherosclerotic plaques (n = 7). **(I)** Plasma BDNF levels measured by ELISA (n = 7). **(J)** Plasma IL-6 and IL-1β levels were quantified by ELISA (n = 7). **(K,** **L)** Representative flow cytometry plots and quantification of CD11b^+^Ly6C^high^ monocytes (n = 7). **(M, N)** Flow cytometry gating strategy and quantification of M1 (F4/80^+^CD86^+^) and M2 (F4/80^+^CD206^+^) BMDMs in model mice (n = 7). **(O)** Representative images of Oil Red O staining of total aortic plaques. Scale bar = 5 mm. **(P)** Analysis of Oil Red O staining to determine the total aortic plaque area (n = 7). **(Q)** Representative images of aortic atherosclerotic plaques. Scale bar = 1 mm. **(R)** Representative images showing the histopathology of aortic sinus plaques. Scale bar = 500 μm. **(S, T)** Plaque size assessments via HE staining and Oil Red O staining (n = 7). **(U)** Necrotic core and collagen fiber area in aortic sinus plaques measured by Masson's trichrome staining (n = 7). The data are presented as the means ± SDs. *P* values were determined using two-sample t tests.

**
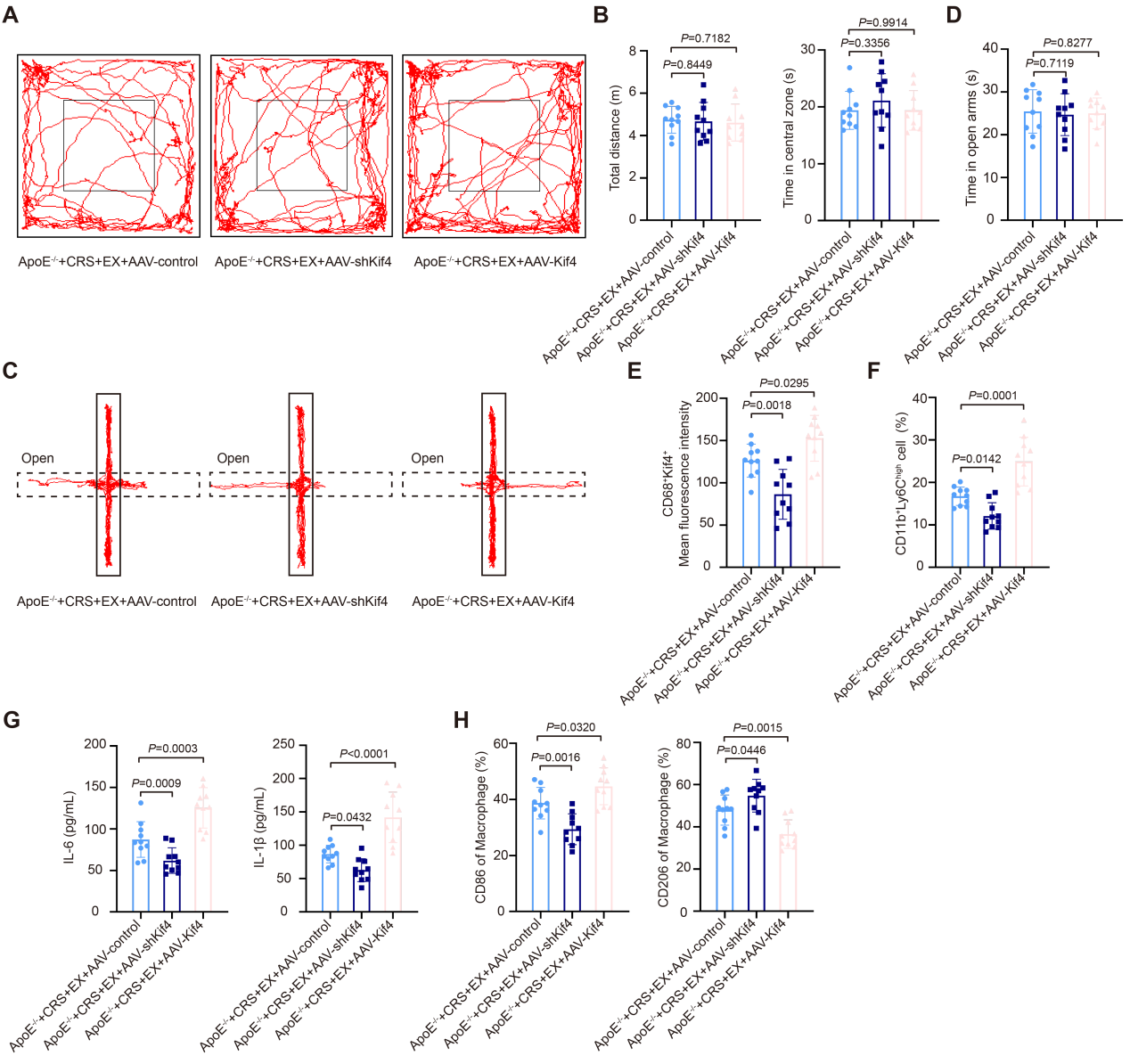
**

**Figure S9. The BDNF–Kif4–TARM1 axis is involved in the exercise-mediated reduction of vascular inflammation and plaque stabilization in model mice. (A)** Representative locomotion tracks of atherosclerotic mice in the open field test (OFT). **(B)** Total distance traveled and time spent in the center zone of the open field by atherosclerotic mice (n = 10). **(C, D)** Representative locomotion tracks in the elevated plus maze (EPM) test and time spent in the open arms of the EPM by model mice (n = 10). **(E)** Quantification of the CD86^+^Kif4^+^ immunofluorescence intensity in atherosclerotic plaques (n = 10). **(F)** Quantification of CD11b^+^Ly6C^high^ monocytes in peripheral blood (n=10). **(G)** Quantification of plasma IL-6 and IL-1β levels by ELISA (n = 10). **(H)** Quantification of M1 (F4/80^+^CD86^+^) and M2 (F4/80^+^CD206^+^) BMDMs from atherosclerotic mice (n = 10). The data are presented as the means ± SDs. *P* values were determined using one-way ANOVA.
