# Supplementary material for: Physical exercise mitigates chronic psychological stress‐induced vascular inflammation via the BDNF–Kif4–TARM1 axis
Source: Clin Transl Med. 2026 Apr 20;16(4):e70674. doi: 10.1002/ctm2.70674 (PMC13096691; doi:10.1002/ctm2.70674)
Supplement: Supplementary file 2 — Supporting Information [file CTM2-16-e70674-s003.docx]

**Supplementary Table**

**Table S1. Baseline characteristics**

| **Characteristics** | **Low-PSS**  **(N=30)** | **Low-PSS+EX**  **(N=30)** | **High-PSS**  **(N=30)** | **High-PSS+EX**  **(N=30)** | ***P* Value** |
| --- | --- | --- | --- | --- | --- |
| **Demographics** | | | |  |  |
| Age, years | 59.50  (49.75, 59.25) | 61.00  (52.00, 69.00) | 57.00  (48.75, 69.00) | 60.50  (50.25, 66.00) | 0.886 |
| Male Sex, n (%) | 24 (80.00) | 23 (76.67) | 20 (66.67) | 21 (70.00) | 0.302 |
| **Medical history, n(%)** | | | |  |  |
| Hypertension | 10 (33.33) | 14 (46.67) | 10 (33.33) | 14 (46.67) | 0.528 |
| Diabetes | 6 (20.00) | 7 (23.33) | 5 (16.67) | 11 (36.67) | 0.287 |
| Previous stroke | 1 (3.33) | 2 (6.67) | 3 (10.00) | 2 (6.67) | 0.784 |
| Current smoker | 11 (36.67) | 10 (33.33) | 7 (23.33) | 4 (13.33) | 0.164 |
| **Clinical profile** | | | |  |  |
| BMI (kg/m^2^) | 24.33±3.24 | 24.43±2.98 | 24.51±2.90 | 24.94±3.42 | 0.883 |
| LVEF (%) | 62.00  (59.75, 63.00) | 61.00  (58.75, 63.00) | 62.00  (58.00, 63.00) | 59.00  (52.75, 63.00) | 0.211 |
| WBC (×10^9^) | 7.49±1.60 | 7.07±1.58 | 8.78±3.01 | 7.32±1.75 | 0.035 |
| Creatinine (μmmol/L) | 80.00  (72.75, 92.00) | 80.50  (71.50, 96.25) | 79.00 (72.75, 90.75) | 76.50  (71.35, 88.00) | 0.872 |
| HbA1c (%) | 6.53±1.62 | 6.99±1.33 | 7.39±1.15 | 6.89±1.14 | 0.125 |
| Total cholesterol (mmol/L) | 3.52±0.71 | 3.64±0.88 | 3.69±1.03 | 3.22±0.90 | 0.171 |
| LDL-C (mmol/L) | 1.80±0.57 | 1.94±0.53 | 1.97±0.52 | 1.65±0.52 | 0.085 |
| HDL-C (mmol/L) | 1.04±0.30 | 1.18±0.96 | 1.04±0.28 | 1.10±0.23 | 0.310 |

Values shown are n (%), mean±SD, or median (interquartile range). BMI, body mass index; LVEF, left ventricular ejection fractions; HbA1c, glycated hemoglobin A1C; LDL-C, low-density lipoprotein cholesterol; HDL-C, high-density lipoprotein cholesterol.
