# Supplementary material for: Physical exercise mitigates chronic psychological stress‐induced vascular inflammation via the BDNF–Kif4–TARM1 axis
Source: Clin Transl Med. 2026 Apr 20;16(4):e70674. doi: 10.1002/ctm2.70674 (PMC13096691; doi:10.1002/ctm2.70674)
Supplement: Supplementary file 3 — Supporting Information [file CTM2-16-e70674-s001.docx]

**Supplemental Materials and Methods**

**2.9 Isolation and culture of bone marrow-derived macrophages (BMDMs)**

BMDMs were flushed from the femurs and tibias of male mice by inserting a needle into the bone cavity and flushing it with DMEM. Macrophages were purified from cell suspensions by centrifugation. Cells were lysed with red blood cell lysis buffer, centrifuged, and resuspended to remove erythrocytes. Primary macrophages were cultured for 7 days at 37°C with 5% CO₂ in Dulbecco’s modified Eagle’s medium (DMEM; Thermo Fisher Scientific) supplemented with 10% FBS (Biological Industries, Israel), 1% penicillin/streptomycin, and macrophage colony-stimulating factor (M-CSF, 30 ng/ml; PeproTech). On day 7, the cells were treated with lipopolysaccharide (LPS; 200 ng/ml; Sigma‒Aldrich) for 24 hours prior to analysis. All the cell cultures tested negative for mycoplasma.

**2.10 Coculture of THP-1 cells and HUVECs**

A direct coculture method was used for the coculture of THP-1 cells and HUVECs. First, HUVECs were seeded into 6-well plates at a density of 10^6^ cells/well. Subsequently, THP-1 cells pretreated with TARM1-Fc (10 µg/mL) or OE-Kif4 were added at a ratio of 5:1 (THP-1 cells to HUVECs). The coculture system was maintained in RPMI-1640 medium supplemented with 10% FBS and 1% penicillin/streptomycin, and the cells were harvested after 24 hours for further analysis.

**2.11 Cell transfection**

Kif4 siRNAs (siKif4-1: 5′–CCAGUCAUCUCGAUCUCAUTT–3′, siKif4-2: 5′–GCUGCAGAUCUUGCUGCUATT–3′, siKif4-3: 5′–CCAGAUACCAGCAGGUCUUTT–3′, and siKif4-4: 5′–GCUGCCAACAAACGUCUUATT–3′) and a nontargeting siRNA (siCtrl) were purchased from Gene Pharma (Suzhou, China). Under appropriate conditions, these siRNAs and siCtrl were transfected into cells with Lipofectamine™ RNAiMAX Transfection Reagent (Invitrogen, USA) following the manufacturer’s instructions. After a 24-hour incubation period, the cells were stimulated with LPS for 24 hours, after which qRT-PCR analysis was performed. The sh-Kif4 plasmid targeting the Kif4 sequence identified from the above experiments was constructed by GenePharma (Suzhou, China). The OE-Kif4 plasmid was constructed by General Biol Co., Ltd. (Anhui, China). Cells were transfected with either the sh-Kif4 plasmid or the OE-Kif4 plasmid using Lipofectamine 3000 (Invitrogen, USA) according to the manufacturer’s instructions. Forty-eight hours later, the cells were stimulated with LPS for 24 hours, followed by subsequent experiments.

**2.12 Generation of knockout cell lines with CRISPR-Cas9**

The deletion of Kil4 in the RAW264.7 cell line was accomplished by Cyagen (Suzhou, China), and that in the THP-1 cell line was accomplished by Ubigene (Guangzhou, China). Briefly, RAW 264.7 or THP-1 cells were transiently transfected with a plasmid carrying the guide DNA for Ki4 and then seeded into a 96-well plate to form monolayers. Knockout clones were screened by PCR and verified by Sanger sequencing.

**2.13 Enzyme-linked immunosorbent assay (ELISA)**

The levels of corticosterone, interleukin-6 (IL-6), interleukin-1β (IL-1β), and BDNF in the plasma of patients and mice were assessed with ELISA kits (corticosterone: CUSABIO, Wuhan, China; IL-6, IL-1β, and BDNF: Jingkang, Shanghai, China) according to the manufacturer’s instructions.

**2.14 Complete blood count determination**

Blood was collected from the mouse retro-orbital plexus, and 100 μL of whole blood was aliquoted into a 1.5-mL EDTA-K_2_-anticoagulated tube. The samples were stored at 4°C prior to analysis. White blood cell (WBC) counts and monocyte counts were determined using a veterinary hematology analyzer (IDEXX, USA).

**2.15 Isolation of peripheral blood monocytes**

Peripheral blood monocytes from patients and mice were isolated using a density gradient technique (TBD, Tianjin, China).

**2.16 Quantitative real-time polymerase chain reaction (qRT-PCR)**

Total RNA was extracted from tissues and cell samples using TRIzol reagent (Invitrogen, Carlsbad, USA). RNA was quantified and reverse transcribed using a PrimeScript RT reagent kit (TOYOBO, Japan) according to the manufacturer’s instructions. Quantitative PCR was performed using qPCR SYBR Green Master Mix (Roche, Basel, Switzerland) on a CFX96^TM^ Real-Time PCR System (Bio-Rad, USA). Relative gene expression was calculated with the comparative threshold cycle method and normalized to β-actin expression. The primer sequences are listed in Supplementary Table S2.

**2.17 Western blot**

The cells were lysed in RIPA lysis buffer supplemented with 1 mM phenylmethylsulfonyl fluoride (PMSF) (Beyotime, China). The cell lysate was centrifuged, and the supernatant was collected as the total protein sample. The protein concentration was determined using a BCA protein assay kit (Beyotime, China). Protein extracts were boiled at 95°C for 10 minutes in 1× loading buffer. Total protein was loaded on an SDS‒PAGE gel, separated, and subsequently transferred to PVDF membranes (Roche Diagnostics GmbH, Mannheim, Germany). After blocking for 1 hour at room temperature with TBST containing 5% skim milk, the membranes were incubated with primary antibodies at 4°C overnight. The membranes were then washed three times with TBST buffer and incubated with the secondary antibody for 1 hour at room temperature. Following three additional washes with TBST buffer, signals were detected using Pierce ECL Western blotting substrate. The protein bands were quantified using Image Lab software. Total, membrane, and cytosolic proteins levels were normalized to those of β-actin (CST, USA), Na^+^/K^+^-ATPase (ABclonal, USA) and β-actin (CST, USA), respectively.

**2.18 Adeno-associated virus injection**

The adeno-associated virus (AAV) vectors AAV-shKif4 and AAV-Kif4 were packaged by PackGene Biotech Co., Ltd. (Guangzhou, China). Each vial of AAV-shKif4 and AAV-Kif4 had a titer of 1 × 10¹³ vector genomes (vg)/ml. In a biosafety cabinet, AAV-shKif4 and AAV-Kif4 were diluted 10-fold in 1 mL of 0.9% saline. Following restraint of the mice, the injection site was disinfected with an alcohol swab. Subsequently, 100 µL of the vector suspension (titer: 1 × 10¹² vg/mL) was administered via tail vein injection using a 1-mL syringe. Chronic psychological stress and/or voluntary wheel running were initiated 10–14 days later.

**2.19 Coimmunoprecipitation (Co-IP) assays**

Following culture, the BMDMs were washed once with phosphate-buffered saline and lysed in prechilled cell lysis buffer containing protease and phosphatase inhibitors. The samples were lysed on ice for 30 minutes, and after centrifugation, the supernatant containing the total protein was collected. The protein concentrations in the cells were measured using a BCA protein assay kit (Beyotime, China). For coimmunoprecipitation, an HA antibody (PTM BioLab, China) was incubated with 50 μl of prewashed protein A/G beads (Selleck, USA) for 15 minutes on a rocking platform. Next, antibody-conjugated beads were collected with a magnet, washed with binding buffer (50 mM Tris, 150 mM NaCl, and 0.1%-0.5% detergent (Triton X-100, Tween 20 or NP40), pH 7.5), and incubated with cell lysates on a rotator overnight at 4°C. The samples were then collected using a magnetic stand and were washed a minimum of three times with binding buffer. After the supernatant was removed, the proteins were eluted with 50 µL of 1×SDS‒PAGE loading buffer, followed by heating at 95°C for 5 minutes. The supernatants were collected for subsequent Western blotting and mass spectrometry analyses.

**2.20 Molecular docking**

The structures of the receptor protein Kif4 and the ligand protein TARM1 were obtained from UniProt (https://www.uniprot.org/). Docking experiments were performed using ZDOCK 3.0.2 software (University of Massachusetts). Following the removal of water molecules, the proteins were subjected to rigid-body docking. The resulting poses were clustered on the basis of ligand-binding site coordinates. The docking results were ranked according to the ZDOCK score. The complex with the highest docking score was selected, visualized using PyMOL molecular graphics system version 2.5.7 (Schrödinger, LLC), and assessed structurally. A negative binding energy change (ΔG < 0) indicates a favorable protein–protein interaction.

**2.21 Isolation of cytoplasmic and membrane proteins**

Cytoplasmic and membrane proteins were isolated using a membrane and cytosol protein extraction kit (Yeasen Biotechnology, Shanghai, China) according to the manufacturer’s instructions. Following the preparation and pretreatment of cells and reagents, the culture medium was removed from the dishes. Solution A was added, and the cells were scraped using a cell scraper. The suspension was subsequently centrifuged at 300 × g for 5 minutes at 4°C. After the supernatant was removed, the pellet was resuspended in Solution A and centrifuged again at 300 × g for 5 minutes. The supernatant was discarded, and the pellet was resuspended in Solution B. This suspension was incubated on a rotary mixer at 4°C for 10 minutes, followed by centrifugation at 16,000 × g for 15 minutes. The resulting supernatant containing cytoplasmic proteins was collected. The cell pellet was resuspended in Solution C and incubated on a rotary mixer at 4°C for 45 minutes. After centrifugation at 16,000 × g for 15 minutes, the supernatant containing the membrane proteins was collected. The extracted cytoplasmic and membrane protein fractions were stored at -80°C and subsequently subjected to Western blot analysis.

**2.22 Histological analysis**

Hearts were harvested from ApoE^-/-^ model mice, fixed with 4% paraformaldehyde at room temperature overnight, and then embedded in optimal cutting temperature compound. Serial cryosections (7–8 μm thick) were prepared using a cryostat (Thermo Fisher Scientific, Waltham, MA, USA). Sections containing the aortic sinus region were mounted for further experiments. The sections were then fixed for 15 minutes with 4% paraformaldehyde and washed three times with PBS. The sections were stained with hematoxylin and eosin (H&E), examined under a light microscope to assess their general morphology, and then imaged. Afterward, the sections were stained with Oil Red O to assess plaque size. Additionally, the sections were subjected to Masson’s trichrome staining according to the manufacturer’s protocol. After staining, the extent of the necrotic core and collagen deposition was quantified under a light microscope. H&E, Masson's trichrome, and Oil Red O staining kits were purchased from Solarbio (Beijing, China). ImageJ software was used to quantify the extent of the plaque lesions.

**2.23 Immunofluorescence staining**

For heart tissues, embedding and sectioning were performed as previously described. Brain tissues were harvested, fixed overnight with 4% PFA at 4°C, and washed with PBS. The tissues were dehydrated through stepwise immersion in 10%, 20%, and 30% sucrose solutions at 4°C until fully saturated. The tissues were then embedded in OCT compound and sectioned at 7–8 μm. For BMDMs, the cells were seeded onto coverslips at an appropriate density and cultured for subsequent experiments. Cells or frozen sections were fixed with 4% paraformaldehyde at room temperature for 15 minutes, permeabilized with 0.1% Triton X-100 in PBS for 30 minutes, blocked with goat serum for 1 hour at room temperature, and subsequently incubated with primary antibodies overnight at 4°C. Following three washes with PBS, the samples were incubated with Alexa Fluor-conjugated secondary antibodies (Invitrogen; 1:500) for 2 hours, after which the nuclei were counterstained with DAPI. Finally, the sections or cells were mounted in anti-fade mounting medium. Fluorescence was observed under a Zeiss LSM800 confocal laser scanning microscope (Carl Zeiss, Inc., Jena, Germany).

**2.24 Adhesion assay**

HUVECs were seeded into a 96-well plate and cultured until they reached complete confluence. In accordance with the manufacturer’s instructions, 2×10⁵ THP-1 cells prestained with 5 μM calcein AM (Beijing, China, C2012) were added to each well and coincubated with the EC monolayer for 30 minutes at 37°C. After the incubation, the nonadherent cells were removed by washing with PBS, and the labeled adherent cells were counted under a fluorescence microscope.

**2.25 Wound healing assay**

After the HUVECs reached confluence, a 200-μL sterile pipette tip was used to create scratch on the cell monolayer, which was then rinsed with phosphate-buffered saline (PBS) to remove the detached cells. Pretreated THP-1 cells were added, and the scratched wound area was observed and imaged to quantify the wound closure rate at 0 hours, 12 hours, and 24 hours.

**2.26 Reactive oxygen species (ROS) detection**

ROS generation was determined using an ROS assay kit (Beyotime, China) according to the manufacturer’s instructions. Briefly, the treated cells were incubated with DMEM containing 10 µmol/L DCFH-DA for 30 minutes at 37°C. After being washed with PBS, the labeled cells were observed using a fluorescence microscope with excitation at 488 nm and emission at 525 nm.

**2.27 JC-1 detection**

The mitochondrial membrane potential was evaluated using a JC-1 kit (Beyotime, China). Treated cells were incubated with the JC-1 solution at 37°C for 20 minutes and then analyzed using a fluorescence microscope with excitation/emission wavelengths of 490/530 nm (for JC-1 monomers, green fluorescence) and 525/590 nm (for JC-1 aggregates, red fluorescence).

**2.28 Chromatin immunoprecipitation‒polymerase chain reaction (ChIP‒PCR)**

A ChIP‒PCR assay was conducted according to the manufacturer's instructions (SimpleChIP Enzymatic Chromatin IP Kit; Cell Signaling Technology, 9003). Four primer pairs targeting the promoter region of Kif4 (designed on the basis of its genomic sequence; see Figure S6E) were synthesized by Sangon Biotech (Shanghai, China) and used to examine the binding of phosphorylated STAT3 (p-STAT3) to the Kif4 promoter via ChIP‒PCR. For ChIP‒PCR, cells were crosslinked with a 37% formaldehyde solution, quenched with a 10× glycine solution, and washed with 1× PBS containing a protease inhibitor cocktail prior to sample processing. Cell nuclei were prepared, and DNA digestion was conducted using micrococcal nuclease according to the manufacturer’s protocol. The lysates were further sonicated for 3 cycles of 20 seconds on and 20 seconds off. The resulting supernatants were incubated with a p-STAT3 antibody (Cell Signaling Technology, USA). A total of 2% of the supernatant was taken as input and stored at -20°C. Each IP sample was added to Protein G Magnetic Beads and incubated at 4°C overnight. The precipitate was repeatedly washed, and ChIP elution buffer was subsequently added, followed by heating at 65°C for 2 hours to elute the chromatin from the antibody/protein G magnetic beads and reverse the cross-links. Finally, the DNA was purified using spin columns and then examined by quantitative real-time polymerase chain reaction.

**2.29 Analysis of single-cell RNA sequencing data**

The gene expression data were obtained from the original article^1^ in the GEO database under accession GSE239591. Filtering and unsupervised clustering were performed using R, including Seurat v.5.1.0.^2^ Specifically, data from the control and HFD groups in the GSE239591 dataset were imported and merged into a single Seurat object. To remove low-quality cells and potential multiplets, the following cell filtering criteria were applied: gene numbers > 200, proportion of UMIs mapped to mitochondrial genes < 10%, proportion of UMIs mapped to hemoglobin genes > 5%, and log10GenesPerUMI > 0.7. The DoubletFinder package v.2.0.3^3^ was subsequently used to identify potential doublets. The FindVariableGenes function was employed to detect the top 2,000 highly variable genes. To obtain normalized gene expression data, the NormalizeData function was used for library size normalization. Principal component analysis (PCA) was performed using the RunPCA function for dimensionality reduction. The FindClusters function was applied to perform graph-based clustering of cells according to their gene expression profiles. Harmony v.1.2.1^4^ was used to remove batch effects from the single-cell RNA sequencing data. Cell visualization was carried out using the 2-dimensional uniform manifold approximation and projection (UMAP) algorithm with the RunUMAP function. The FindAllMarkers function was used to identify marker genes for each cluster. The cell type information in this dataset was derived from the original article. TARM1 gene expression was subsequently examined.

**Table S2. Sequence of primers in real-time qPCR.**

| **Genes** | **Primer** | **Sequence（5’- 3’）** |
| --- | --- | --- |
| IL-1β_MOUSE | Forward | GCTTCAGGCAGGCAGTATC |
|  | Reverse | ATGGGCTCTTCTTCAAAG |
| IL-6_MOUSE | Forward | TAGTCCTTCCTACCCCAATTTCC |
|  | Reverse | TTGGTCCTTAGCCACTCCTTC |
| Nos2_MOUSE | Forward | AGCCCTCACCTACTTCCTG |
|  | Reverse | TCTCTGCCTATCCGTCTC |
| CCL2_MOUSE | Forward | CACTCACCTGCTGCTACTCATTC |
|  | Reverse | TCTTTGGGACACCTGCTG |
| TNF-α_MOUSE | Forward | GGTTCTGTCCCTTTCACTCAC |
|  | Reverse | CTCTTCTGCCAGTTCC |
| Arg1_MOUSE | Forward | CTCCAAGCCAAAGTCCTTAGAG |
|  | Reverse | AGGAGCTGTCATTAGGGACATC |
| CD206_MOUSE | Forward | CTCTGTTCAGCTATTGGACGC |
|  | Reverse | CGGAATTTCTGGGATTCAGCTTC |
| Retnlb_MOUSE | Forward | AAGCCTACACTGTGTTTCCTTTT |
|  | Reverse | GCTTCCTTGATCCTTTGATCCAC |
| MGL_MOUSE | Forward | TGAGAAAGGCTTTAAGAACTGGG |
|  | Reverse | GACCACCTGTAGTGATGTGGG |
| Kif4_MOUSE | Forward | AGAGCAGGGTAACAACTCCAG |
|  | Reverse | CCAGCGAGGTCTACAAGATGC |
| Lhfpl2_MOUSE | Forward | ATGAGCAGGCAGGCATGAAC |
|  | Reverse | CAGAAGCCGCTGGCTATCTC |
| IL-10_HUMAN | Forward | GTTGTTAAAGGAGTCCTTGCTG |
|  | Reverse | TTCACAGGGAAGAAATCGATGA |
| CD163_HUMAN | Forward | CATTATGTCCTTCAGAGCAAGTG |
|  | Reverse | AGCGACCTCCTCCATTTACC |
| ICAM-1_MOUSE | Forward | ATGCCCAGACATCTGTGTCC |
|  | Reverse | GGGGTCTCTATGCCCAACAA |
| VCAM-1_MOUSE | Forward | GGGAAGATGGTCGTGATCCTT |
|  | Reverse | TCTGGGGTGGTCTCGATTTTA |
| BDNF_MOUSE | Forward | TCATACTTCGGTTGCATGAAGG |
|  | Reverse | AGACCTCTCGAACCTGCCC |
| β-actin_MOUSE | Forward | ACGGCCAGGTCATCACTATTG |
|  | Reverse | CAAGAAGGAAGGCTGGAAAAGA |
| Kif4_HUMAN | Forward | TACTGCGGTGGAGCAAGAAG |
|  | Reverse | CATCTGCGCTTGACGGAGAG |
| β-actin_HUMAN | Forward | TCATGAAGTGTGTGACGTGGACATC |
|  | Reverse | CAGCAGGAGCAATGATCTTGATCT |

**Table S3. List of key reagents and sources.**

| **Antibody** | | | | | |  |
| --- | --- | --- | --- | --- | --- | --- |
| **Reagent or Resource name** | **Provider** | | **Application** | **Working Concentration** | | **Cat# or identifier#** |
| FITC anti-mouse CD45 | BioLegend | | FC | 0.5 μl/test | | 103107 |
| APC anti-mouse/human CD11b | BioLegend | | FC | 1.25 μl/test | | 101211 |
| PE anti-mouse Ly-6C | BioLegend | | FC | 1.25 μl/test | | 128007 |
| PE anti-mouse CD86 | Biolegend | | FC | 3 μl/test | | 150007 |
| APC anti-mouse CD206 | Invitrogen | | FC | 1.25 μl/test | | 141707 |
| FITC anti-mouse F4/80 | Biolegend | | FC | 0.5 μl/test | | 123107 |
| APC anti-human CD163 | Biolegend | | FC | 5 μl/test | | 333610 |
| FITC anti-human CD68 | Biolegend | | FC | 5 μl/test | | 333806 |
| PE anti-human CD86 | Biolegend | | FC | 5 μl/test | | 374206 |
| Anti-CD68 | Abcam | | IF | 1:500 | | ab53444 |
| Anti-β-actin | CST | | WB | 1:2000 | | 3495T |
| Anti-Na^+^K^+^ATPase | ABclonal | | WB | 1:100 | | 14418-1-AP |
| Anti-Kif4 | Proteintech | | WB/IF | 1:2000/1:100 | | 14344-1-AP |
| Anti-Tarm1 | Santa | | WB/IF | 1:500/1:100 | | sc-514218 |
| Anti-Tarm1 | Stjohnslabs | | WB | 1:1000 | | STJ194662 |
| Anti-ICAM-1 | Proteintech | | WB | 1:1000 | | 10831-1-AP |
| Anti-VCAM-1 | PTM | | WB | 1:1000 | | PTM-6623 |
| Anti-STAT3 | CST | | WB | 1:2000 | | 4904S |
| Anti-p-STAT3 | CST | | WB | 1:2000 | | 9145S |
| Anti-TrkB | Abcam | | WB | 1:500 | | ab187041 |
| Anti-p-TrkB | absin | | WB | 1:1000 | | abs140218 |
| Anti-HA | PTM | | WB | 1:5000 | | PTM-5389 |
| Anti-α-Tubulin | PTM | | IF | 1:100 | | PTM-5442 |
| Anti-BDNF | Abcam | | IF | 1:100 | | ab108319 |
| DAPI | Invitrogen | | IF | 1ug/ml | | D1306 |
| Goat Anti-rabbit IgG H&L (Alexa Fluor® 594) | Abcam | | IF | 1:200 | | ab150080 |
| Goat Anti-Rat IgG H&L (Alexa Fluor® 488) | Abcam | | IF | 1:200 | | ab150157 |
| [Goat Anti-Mouse IgG H&L (Alexa Fluor® 488)](https://www.abcam.cn/products/secondary-antibodies/goat-mouse-igg-hl-alexa-fluor-594-ab150116.html) | Abcam | | IF | 1:200 | | ab150113 |
| HRP-Conjugated Goat Anti-Rabbit IgG | ZSGB-BIO, Beijing, China | | WB | 1:10000 | | ZB-5301 |
| HRP-Conjugated Goat Anti-Mouse IgG | ZSGB-BIO, Beijing, China | | WB | 1:1000 | | ZB-2305 |
| Donkey Anti-Goat IgG H&L (Alexa Fluor® 647) | Abcam | | IF | 1:200 | | ab150131 |
| PE anti-human CD14 | BioLegend | | FC | 5 μl/test | | 301805 |
| FITC anti-human CD16 | BioLegend | | FC | 5 μl/test | | 302005 |
| **Critical Commercial Assay Kits** | | | | | | |
| **Reagent or Resource name** | | **Provider** | | | **Cat# or identifier#** | |
| Mouse Peripheral Blood Monocytes Isolation Kit | | TBD, Tianjin, China | | | TBD2011M | |
| Human Peripheral Blood Monocytes Isolation Kit | | TBD, Tianjin, China | | | TBD2011H05 | |
| Mouse IL-6 ELISA Kit | | Jiangkang, Shanghai, China | | | JLC-JK221 | |
| Mouse IL-1β ELISA Kit | | Jiangkang, Shanghai, China | | | JLC-JK003 | |
| Mouse BDNF ELISA Kit | | Jiangkang, Shanghai, China | | | JLC4037 | |
| Human BDNF ELISA Kit | | Jiangkang, Shanghai, China | | | JLC7233 | |
| Mouse Corticosterone ELISA Kit | | CUSABIO, Wuhan, China | | | CSB-E07969m | |
| Pierce Cell Surface Protein Isolation Kit | | Thermo Scientific™ | | | 89881 | |
| SimpleChIP Enzymatic Chromatin IP Kit | | CST | | | #9003 | |
| Membrane and Cytoplasmic Protein Extraction Kit | | Beyotime, Shang, China | | | P0033 | |
| Membrane and Cytoplasmic Protein Extraction Kit | | Yanmei, Shanghai, China | | | PC202S | |
| H&E Staining Kit | | Solarbio, Beijing, China | | | G1120 | |
| Modified Masson's Trichrome Staining Kit | | Solarbio, Beijing, China | | | G1346 | |
| Modified Oil Red O Staining Kit | | Solarbio, Beijing, China | | | G1261 | |
| Transcriptor First Strand cDNA Synthesis Kit | | Roche | | | 11483188001 | |
| ECL Ultra-Sensitive Detection Kit | | HaiGene, Harbin, China | | | M2301 | |
| FastStart Universal SYBR Green Master Kit (ROX) | | Roche | | | 04913914001 | |
| Reactive Oxygen Species (ROS) Assay Kit | | Beyotime, Shang, China | | | S0033S | |
| JC-1 Mitochondrial Membrane Potential Assay Kit | | Beyotime, Shang, China | | | C2006 | |
| **Other key reagents** | | | | | | |
| **Reagent or Resource name** | | **Provider** | | | **Cat# or identifier#** | |
| TARM1-Fc | | Sino Biological, Beijing, China | | | N/A | |
| Recombinant Human/Murine/Rat BDNF | | PeproTech | | | 450-02 | |

**REFERENCES**

1. Mazan-Mamczarz K, Tsitsipatis D, Childs BG, et al. Single-cell and spatial transcriptomics map senescent vascular cells in arterial remodeling during atherosclerosis in mice. *Nat Aging*. 2025;5(8):1528-1547.

2. Hao Y, Hao S, Andersen-Nissen E, et al. Integrated analysis of multimodal single-cell data. *Cell*. 2021;184(13):3573-3587 e29.

3. McGinnis CS, Murrow LM, Gartner ZJ. DoubletFinder: Doublet Detection in Single-Cell RNA Sequencing Data Using Artificial Nearest Neighbors. *Cell Syst*. 2019;8(4):329-337 e4.

4. Korsunsky I, Millard N, Fan J, et al. Fast, sensitive and accurate integration of single-cell data with Harmony. *Nat Methods*. 2019;16(12):1289-1296.
